# PCRCR complex is essential for invasion of human erythrocytes by *Plasmodium falciparum*

**DOI:** 10.1038/s41564-022-01261-2

**Published:** 2022-11-17

**Authors:** Stephen W. Scally, Tony Triglia, Cindy Evelyn, Benjamin A. Seager, Michał Pasternak, Pailene S. Lim, Julie Healer, Niall D. Geoghegan, Amy Adair, Wai-Hong Tham, Laura F. Dagley, Kelly L. Rogers, Alan F. Cowman

**Affiliations:** 1 The Walter and Eliza Hall Institute of Medical Research, Parkville, Australia; 2 University of Melbourne, Melbourne, Australia

**Keywords:** Parasite biology, X-ray crystallography

## Abstract

The most severe form of malaria is caused by *Plasmodium falciparum*. These parasites invade human erythrocytes, and an essential step in this process involves the ligand PfRh5, which forms a complex with cysteine-rich protective antigen (CyRPA) and PfRh5-interacting protein (PfRipr) (RCR complex) and binds basigin on the host cell. We identified a heteromeric disulfide-linked complex consisting of *P. falciparum* Plasmodium thrombospondin-related apical merozoite protein (PfPTRAMP) and *P. falciparum* cysteine-rich small secreted protein (PfCSS) and have shown that it binds RCR to form a pentameric complex, PCRCR. Using *P. falciparum* lines with conditional knockouts, invasion inhibitory nanobodies to both PfPTRAMP and PfCSS, and lattice light-sheet microscopy, we show that they are essential for merozoite invasion. The PCRCR complex functions to anchor the contact between merozoite and erythrocyte membranes brought together by strong parasite deformations. We solved the structure of nanobody–PfCSS complexes to identify an inhibitory epitope. Our results define the function of the PCRCR complex and identify invasion neutralizing epitopes providing a roadmap for structure-guided development of these proteins for a blood stage malaria vaccine.

## Abstract (web summary)

A heteromeric disulfide-linked complex binds to the RCR complex and is required for invasion of human erythrocytes by *Plasmodium falciparum*.

## Main

Invasion of erythrocytes by the malaria-causing parasite *Plasmodium* *falciparum* is complex and involves an initial interaction of merozoites followed by attachment and internalization^1^. Merozoite interaction with the erythrocyte involves wrapping and deformation of the host cell membrane to orientate the parasite so its apical end binds the erythrocyte membrane via ligand–receptor interactions^2^. A moving junction is formed between the merozoite and erythrocyte membrane and propelled along the surface of the parasite cell to the posterior end via force generated by the parasite actomyosin motor followed by fusion of the host membrane and parasite internalization^1,3–5^.

PfRh5 binds to the receptor basigin^6^ and functions with cysteine-rich protective antigen (CyRPA)^7–9^ and PfRh5-interacting protein (PfRipr)^10^ to form the RCR complex^9,11^. The RCR complex is linked to a Ca^2+^ flux occurring during merozoite invasion^9,12^ and inserts into the membrane of the erythrocyte during invasion^11^. Other *Plasmodium* species infecting humans do not have an orthologue of PfRh5 and consequently are not dependent on basigin for merozoite invasion^13^. Plasmodium thrombospondin-related apical merozoite protein (PTRAMP) and cysteine-rich small secreted protein (CSS) in *Plasmodium* *knowlesi* form a trimeric complex with PkRipr, the homologue of PfRipr^13^. PkPTRAMP binds to erythrocytes, and it has been hypothesized that this protein provides the means for PkCSS–PkPTRAMP–PkRipr complex binding to erythrocytes, thus performing a function equivalent to PfRh5 in *P. falciparum*^13^.

In this Article, we identified PfPTRAMP and PfCSS as interacting partners of the RCR complex in *P. falciparum*. The PfPTRAMP–PfCSS–PfRipr–CyRPA–PfRh5 (PCRCR) complex is essential for merozoite invasion. We identified nanobodies to PfCSS and PfTRAMP that block merozoite invasion, and the three-dimensional structure of nanobody–PfCSS complexes were determined to identify neutralizing epitopes. Using lattice light-sheet microscopy (LLSM) of merozoites lacking PfRh5, PfPTRAMP and PfCSS, we determined the function of PCRCR.

## Results

### PfPTRAMP and PfCSS are essential for invasion

**Fig. 1:**
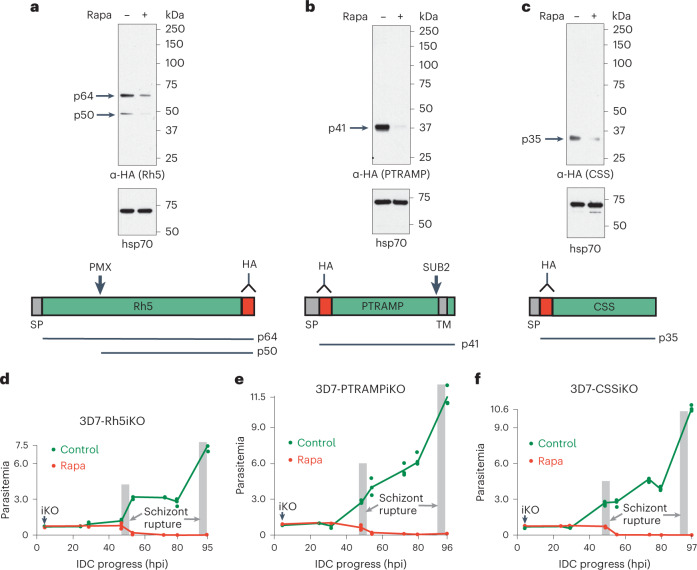
PfPTRAMP, PfCSS and PfRh5 are essential for growth of *P. falciparum*. **a**–**c**, Inducible knockdown of PfRh5 (**a**), PfPTRAMP (**b**) and PfCSS (**c**) expression. Rapa minus and plus rapamycin. HA-tagged PfRh5, PfPTRAMP and PfCSS were detected using anti-HA antibodies. Molecular weight markers (kDa) are shown on the right. Below each panel is a diagram of the protein with the position of the HA tag (red) marked with an antibody symbol. The relevant PMX and SUB2 protease cleavage sites are shown for PfRh5^40^. Signal peptide sequence (SP) at N-terminus and transmembrane sequence (TM) are grey. The predicted (p) size of each processed polypeptide is shown. **d**–**f**, Representative experiments showing *P. falciparum* parasitemia over time plus (red lines) and minus (green lines) rapamycin for inducible knockdown of PfRh5 (3D7–Rh5iKO) (**d**), PfPTRAMP (3D7–PTRAMPiKO) (**e**) and PfCSS (3D7–CSSiKO) (**f**). Intraerythrocytic developmental cycle (IDC). Hours post invasion (hpi). Also shown in Extended Data Fig. 1g–i is a second independent representative experiment. Source data

**Fig. 2:**
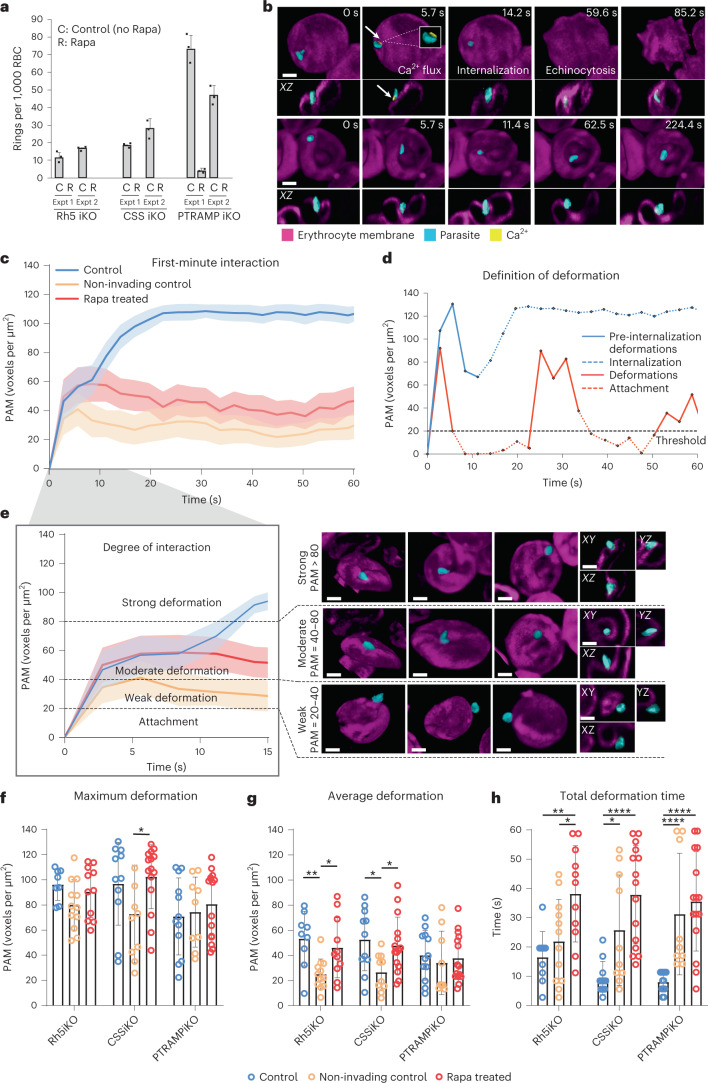
Conditional knockout of PfRh5, PfPTRAMP and PfCSS shows their function was essential for invasion of human erythrocytes by *P. falciparum* merozoites. **a**, Quantitation of merozoite invasion of erythrocytes when expression of PfRh5, PfPTRAMP or PfCSS was knocked down using rapamycin. Shown are each *P. falciparum* parasite line in which either PfRh5 (3D7–PfRh5iKO), PfPTRAMP (3D7–PTRAMPiKO) or PfCSS (3D7–CSSiKO) is under inducible knockout control with rapamycin (R) compared with control with no rapamycin (C). Histogram represents two independent experiments (Expt 1 and Expt 2) of each parasite line with mean ± s.e.m. **b**, Representative snapshots showing parasites (cyan) interacting with erythrocytes (magenta) pre-loaded with Ca^2+^ indicator (yellow) displayed using Imaris in 3D Blend mode and *XZ* views. In the control condition (top; 3D7–PfRh5iKO), the parasite shows a Ca^2+^ flux with internalization and echinocytosis. In rapamycin-treated (Rapa) (bottom; 3D7–PfRh5iKO), the parasite caused deformations on the erythrocyte but no invasion or echinocytosis. Scale bars, 2 μm. **c**, PAM time plot showing the first-minute interaction by invading control (blue, *n* = 32), non-invading control (orange, *n* = 31) and Rapa-treated parasites (red, *n* = 41) with neighbouring erythrocytes after parasite egress, where *t* = 0 represents the timepoint immediately before interaction began. Solid lines represent mean values, and shaded regions represent ±95% confidence interval (CI). **d**, PAM time plot from two parasite–erythrocyte interactions showing the definition of deformation (solid red and blue lines), which exclude the internalization period and beyond (dashed blue line), as well as periods where PAM ≤ 20 voxels per μm^2^ (dashed red lines). **e**, PAM time plot from **c** labelled with thresholds for defining the degree of parasite–erythrocyte interaction and respective images for visualization. Images show three examples in 3D Blend mode view and one example in *XY*, *YZ* and *XZ* views each for weak, moderate and strong deformations, displayed with Imaris. Scale bars, 2 μm. **f**–**h**, Bar graphs showing maximum deformation (*P = 0.0308) (**f**), average deformation (*P* values from left to right: **P = 0.0043, *P = 0.0268, *P = 0.0197, *P = 0.0137) (**g**) and total deformation time (*P* values from left to right: **P = 0.0051, *P = 0.0233, ****P < 0.0001, *P = 0.0155, ****P < 0.0001, ****P < 0.0001) (**h**) during the first-minute interaction by invading control (blue), non-invading control (orange) and Rapa-treated (red) parasites from 3D7–PfRh5iKO (*n* = 9 for control, *n* = 12 for non-invading control, *n* = 11 for Rapa-treated), 3D7–CSSiKO (*n* = 11 for control, *n* = 10 for non-invading control, *n* = 15 for Rapa-treated) and 3D7–PTRAMPiKO (*n* = 12 for control, *n* = 9 for non-invading control, *n* = 15 for Rapa-treated) parasite lines. Bar heights represent mean values, and error bars represent standard deviation (s.d.). Mann–Whitney two-tailed test. Source data

**Extended Data Fig. 1:**
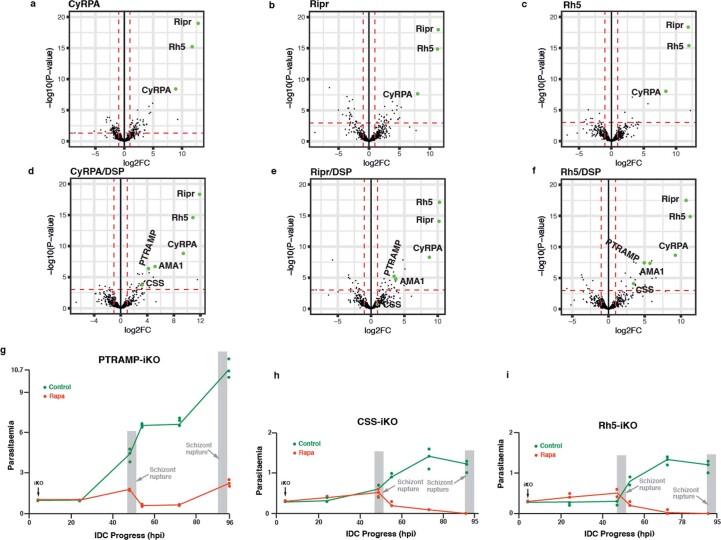
PfPTRAMP and PfCSS interact with the PfRh5 complex and play an essential role for growth of *P. falciparum*. **a-f**. Volcano plots illustrating the log2 fold change (log2FC) of immunoprecipitated HA-tagged CyRPA, PfRipr, or PfRh5 versus 3D7 before and after cross-linking with DSP versus significance of the change (-log10 P value). Differential protein expression analysis was performed using Limma which involves a moderated t-test. Benjamini and Hochberg’s method was used to adjust the p-values for multiple testing. Proteins were deemed differentially regulated in the log2 fold change in protein expression was 1-fold and exhibited an adjusted p-value ≤ 0.05. Proteins that were significantly immunoprecipitated with PfCyRPA-HA, PfRipr-HA and PfRh5-HA were analysed further and this included PfPTRAMP, PfCSS and Apical Membrane Protein 1 (AMA1). In this study we concentrated on PfPTRAMP and PfCSS. We are testing a potential link of AMA1 with the function of the PCRCR complex and this will be published elsewhere. An additional protein that was significantly found in all three immunoprecipitation experiments was heat shock protein 70 (PF3D7_0917900) but this was not considered further because it is a highly expressed chaperone protein and frequently immuno-precipitates in experiments such as these. Proteins that immunoprecipitated significantly in less than the three conditions were analysed with respect to subcellular location, timing of expression and potential role in merozoite invasion and because they did not match these criteria were not analysed further. **a**. Immuno-precipitated HA-tagged CyRPA. **b**. Immunoprecipitated HA-tagged PfRipr. **c**. Immuno-precipitated HA-tagged PfRh5. **d**. Immunoprecipitated HA-tagged CyRPA after cross-linking proteins with DSP. **e**. Immuno-precipitated HA-tagged PfRipr after cross-linking proteins with DSP. **f**. Immuno-precipitated HA-tagged PfRh5 after cross-linking proteins with DSP. **g**. Parasitemia of *P. falciparum* parasites with rapamycin inducible knockdown of PfPTRAMP. **h**. Parasitemia of *P. falciparum* parasites with rapamycin inducible knockdown of PfCSS. **i**. Parasitemia of *P. falciparum* parasites with rapamycin inducible knockdown of PfRh5. Source data

**Extended Data Fig. 2:**
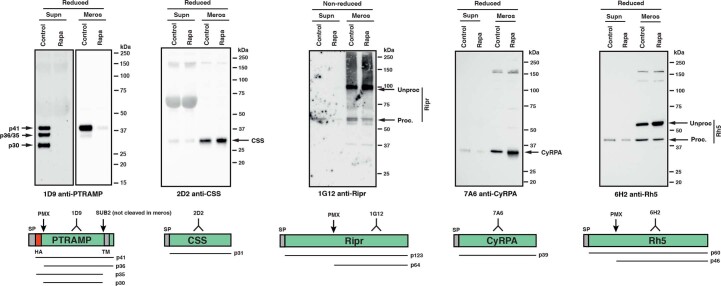
PfCSS, PfRipr, CyRPA and PfRh5 are expressed at normal levels when PfPTRAMP expression is removed by conditional gene knockout. 3D7-PTRAMPiKO was grown without (Control) and with rapamycin (Rapa) to mature schizonts and merozoites and supernatants prepared and analysed by SDS-PAGE and immunoblots that were probed with mAbs, 1D9 (PfPTRAMP), 2D2 (PfCSS), 1G12 (PfRipr), 7A6 (CyRPA) and 6H2 (PfRh5). At bottom of each panel is structure of the relevant protein with the domain recognised by each mAb with respect to PMX protease processing. The PfPTRAMP protein had a HA tag towards the N-terminus (red). The grey domains correspond to the Signal Sequence (SP). The molecular weight of the processed polypeptides and position within each protein is shown. Supernatants were prepared from purified schizonts placed back in culture and allowed to egress. Merozoites were centrifuged as pellets and prepared for SDS-PAGE analysis. Source data

Immunoprecipitation of PfRh5, CyRPA and PfRipr from *P. falciparum* and analysis using mass spectrometry identified PfPTRAMP^14^ and PfCSS^15^ as components of the RCR complex (Extended Data Fig. 1a–f). To analyse the function of PfPTRAMP, PfCSS and PfRh5, each corresponding gene (*pfptramp*, PF3D7_1218000, *pfcss*, PF3D7_1404700 and *pfrh5*, PF3D7_0424100) was placed under conditional control^15^. Conditional deletion of *Pfptramp*, *pfcss* and *pfrh5* resulted in substantial decreases in protein expression (Fig. 1a–c). It was also shown that expression levels of PfRh5, CyRPA and PfRipr were not affected by the knockdown of PfTRAMP or PfCSS (Extended Data Fig. 2). Analysis of parasite growth showed they were not able to expand, indicating the function of each protein was essential (Fig. 1d–f and Extended Data Fig. 1g–i) and that the schizont to ring stage transition was blocked, consistent with these proteins being required for invasion (Fig. 2a).

### PCRCR captures surface contact of merozoite and erythrocyte

PfPTRAMP, PfCSS and PfRh5 function was analysed using LLSM to quantitate interaction of merozoites with erythrocytes (Fig. 2b–h and Supplementary Table 1)^9,12,16^. 3D7–PTRAMPiKO, 3D7–CSSiKO and 3D7–Rh5iKO grew efficiently and invaded erythrocytes (Fig. 2b)^1,9,12^. The interactions of 21 merozoites were visualized for each parasite (63 observations), and of these 32 merozoites (9, 12 and 11 for each parasite line) successfully invaded with most showing a Ca^2+^ flux (Fig. 2b, Supplementary Table 1 and Supplementary Video 1). The ~50% invasion frequency for merozoites interacting with erythrocytes accords well with previous studies^1,9,12^.

3D7–Rh5iKO, 3D7–PTRAMPiKO and 3D7–CSSiKO were grown in rapamycin and merozoites imaged interacting with erythrocytes. Between 11 and 15 merozoites were imaged for each parasite line (41 observations) (Supplementary Table 1), and of these none invaded (Fig. 2b and Supplementary Video 2). The phenotype observed was identical for PfPTRAMP, PfCSS and PfRh5 conditional knockout merozoites. Therefore, PfPTRAMP and PfCSS are essential and presumably function at the same step as PfRh5 and the RCR complex in merozoite invasion^9,12^.

To characterize merozoite–erythrocyte interactions, we developed a semi-automated method for quantitation of surface contact between the parasite and host cell that we termed parasite-associated host membrane (PAM), which was used to quantitate deformations (Fig. 2c–h). PAM of 3D7–Rh5iKO, 3D7–PTRAMPiKO and 3D7–CSSiKO merozoites during invasion increased rapidly over the first 10 s (Fig. 2c), and this defined host membrane deformations (Fig. 2d). After 10–25 s the magnitude of PAM showed a second phase of increase and reached a level where the parasite became fully wrapped, which was maintained after internalization. The plateauing between the end of deformation and internalization was consistent with parasite ‘recoiling’, often concurrent with Ca^2+^ flux^16^. This ‘recoil’ phase was a dip in PAM before internalization, in the example of single merozoite interactions (Fig. 2d). However, this feature was obscured in averaged PAM from multiple merozoites due to timing variabilities for pre-internalization deformations and recoiling (Fig. 2c).

In contrast, rapamycin-treated 3D7–Rh5iKO, 3D7–PTRAMPiKO and 3D7–CSSiKO merozoites had a significantly decreased PAM (surface area) of the parasite interacting with the host membrane and an extended period of moderate to weak deformations (Fig. 2c–e). During the first 10 s of interaction, deformation was the same as parental merozoites. The magnitude of deformation then dipped significantly after the first 10 s, went through rounds of increasing and decreasing surface contact (PAM) and in some cases fell below the deformation threshold as the parasite continued to make unsuccessful attempts to invade (Fig. 2d,e). Rapamycin-treated merozoites displayed similar maximum and average deformations compared with invading parental merozoites (Fig. 2f,g). However, total deformation time showed a highly significant increase for merozoites lacking PfRh5, PfPTRAMP or PfCSS function (Fig. 2h). In the absence of the function of these proteins, the parasite rebounds from the strong deformation to a baseline with a minimal degree of PAM at the apical end. Therefore, the function of PfRh5, PfPTRAMP and PfCSS is not required for establishment and maintenance of the initial merozoite apical interaction with the erythrocyte that precedes and is required for strong deformations. However, it is required to capture and hold the increased membrane surface contact formed between the merozoite and erythrocyte membranes created by strong deformations. In addition, merozoites are capable of multiple rounds of strong deformations mediated by generation of force from the parasite pushing into the host cell membrane.

### PfPTRAMP and PfCSS are present on invading merozoites

**Fig. 3:**
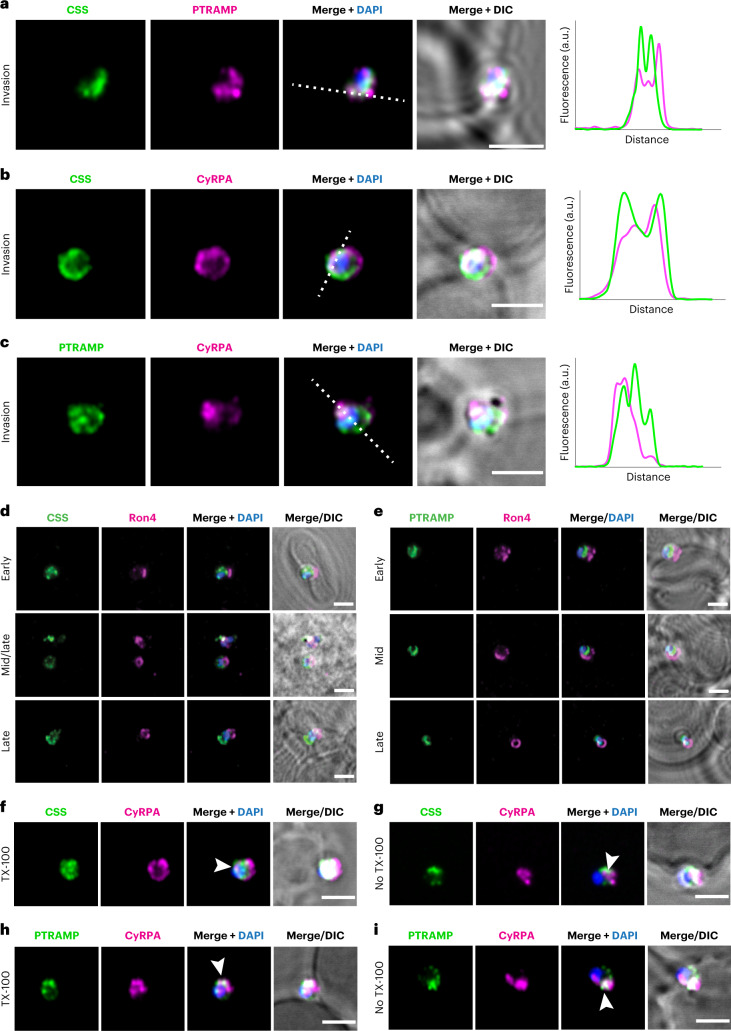
PfCSS and PfPTRAMP co-localize with each other and with CyRPA during invasion. Super-resolution imaging of invading merozoites. **a**, PfCSS was detected using an HA antibody, and PfPTRAMP was detected using the 3D8 mouse monoclonal antibody. Both proteins overlap during merozoite invasion. **b**,**c**, HA-tagged PfCSS **(b)** and PfPTRAMP **(c)** were co-stained with CyRPA and overlapped during merozoite invasion. Scale bars, 2 µm. Intensity plots along the drawn dashed line are displayed on the right side. **d**,**e**, Invading merozoites were fixed and stained for PfCSS–HA (**d**) or HA–PfPTRAMP (**e**) together with RON4. RON4 was used as a marker of the tight junction and allowed to differentiate between early, mid, and late invasion events. **f**–**i**, PfCSS–HA (**f**,**g**) or HA–PfPTRAMP (**h**,**i**) invading merozoites were fixed and either permeabilized (TX-100, **f**,**h**) or not (no TX-100, **g**,**i**) before staining for HA and CyRPA. Positive signal in the absence of permeabilization suggests that the labelled proteins are exposed at this stage, allowing for the access of antibodies. Arrows show signal overlap. 4′,6-diamidino-2-phenylindole (DAPI). Differential interference contrast (DIC). Scale bars, 2 µm.

Subcellular localization of PfPTRAMP and PfCSS during merozoite invasion was determined and compared with the RCR complex^9^. PfPTRAMP and PfCSS were concentrated at the merozoite apical end abutting the erythrocyte membrane during invasion with strong overlap in co-localization with each other and CyRPA; however, there were areas with weaker overlap (Fig. 3a–c). To determine the subcellular localization of PfCSS and PfPTRAMP during merozoite invasion, co-localization experiments were performed with RON4. RON4 shows a ring fluorescence surrounding the parasite that corresponds to the moving junction^17^. PfCSS and PfPTRAMP localization was posterior to RON4 in the merozoite (Fig. 3d,e) consistent with a surface location where they would be removed by SUB2 sheddase as the junction extends to the posterior of the merozoite during invasion^18^. PfCSS and PfPTRAMP were located on the surface of invading merozoites as shown using Triton X-100 (TX-100)-treated and untreated parasites (Fig. 3f–i).

### PfPTRAMP forms a disulfide bonded heterodimer with PfCSS

**Fig. 4:**
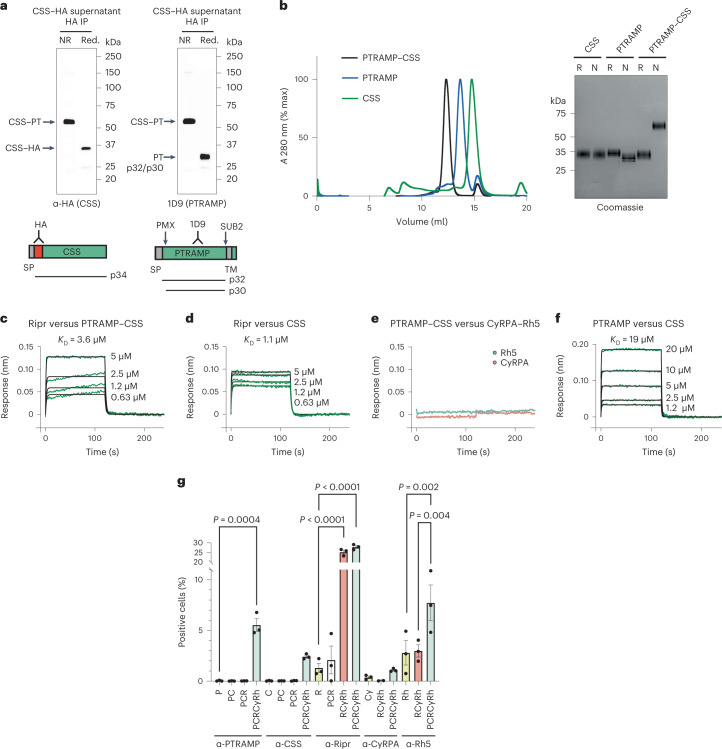
PfPTRAMP and PfCSS form a functional heterodimer and a complex with PfRipr, CyRPA and PfRh5 to enhance PfRh5 binding to erythrocytes. **a**, The *P. falciparum* line PfCSS–HA was used for immunoprecipitation (IP) from merozoite supernatants of PfCSS using anti-HA antibodies (left) and PfPTRAMP using monoclonal antibody 1D9 (right) under non-reduced (NR) and reduced (Red.) conditions. The positions of the PTRAMP–CSS heterodimer, PfCSS–HA and PfPTRAMP (p32 and p30) proteins detected are arrowed. Shown are cartoons of PfCSS and PfPTRAMP with the position of antibody epitopes, the processing by PMX and SUB2 and the polypeptides detected. **b**, Size exclusion chromatography profiles for PTRAMP–CSS (black), PfPTRAMP (blue) and PfCSS (green) from a Superdex 200 Increase 10/300 GL column. Absorbance (A). SDS–PAGE of the final purified PfPTRAMP, PfCSS and PTRAMP–CSS proteins in reducing (R) and non-reducing (N) conditions. The molecular weight markers are shown on the left in kDa. **c**–**f**, Representative sensorgrams and 1:1 model best fit (black). **c**, PfRipr binding to PTRAMP–CSS (Ripr versus PTRAMP–CSS). **d**, PfRipr binding to PfCSS (Ripr versus CSS). **e**, PTRAMP–CSS does not bind to PfRh5 or CyRPA (PTRAMP–CSS versus Rh5 and CyRPA). **f**, PfPTRAMP binding to PfCSS (PTRAMP versus CSS). **g**, Fluorescence-Activated Cell Sorting (FACS) analysis of different combinations of PfPTRAMP (P), PfCSS (C), PfRipr (R), CyRPA (Cy) and PfRh5 (Rh) binding to erythrocytes. *N* = 3; experiments were performed at least 3 times with biologically independent samples and were reproducible. Error bars represent s.e.m. Statistical significance was determined by an ordinary one-way analysis of variance with Tukey’s multiple comparisons test. Exact *P* values are shown in the figure where applicable. Source data

**Extended Data Fig. 3:**
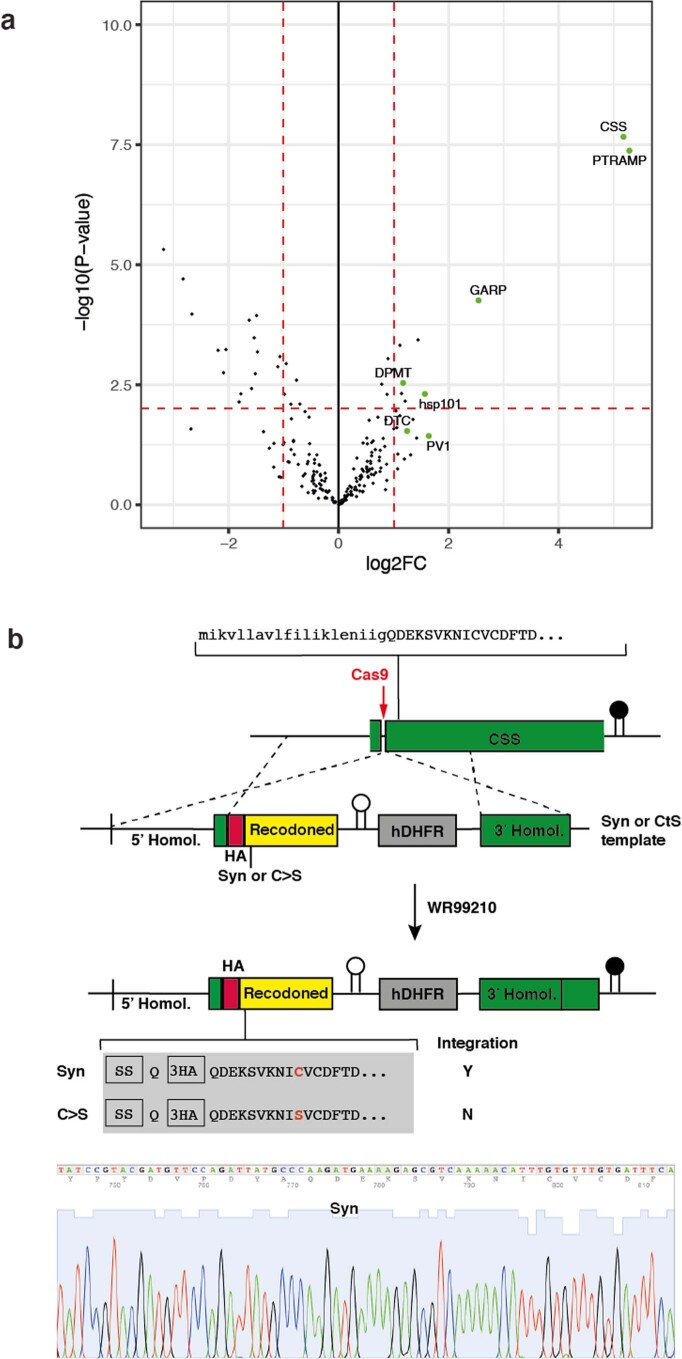
PfPTRAMP and PfCSS form a complex and the PfCSS C30 residue appears to be essential. **a**. Volcano plot illustrating the log_2_ protein ratios of immunoprecipitated PfCSS-HA proteins versus 3D7 control as analysed by mass spectrometry analysis. Differential protein expression analysis was performed using Limma which involves a moderated t-test. Benjamini and Hochberg’s method was used to adjust the p-values for multiple testing. Proteins were deemed differentially regulated in the log2 fold change in protein expression was 1-fold and exhibited an adjusted *p*-value ≤ 0.05. N = 3 biologically independent samples used. **b**. Scheme to construct *P. falciparum* lines that express PfCSS with the amino acid Cys30 mutated to Ser30 using CRISPR. Shown is the Cas9 cleavage site (red) near the protospacer adjacent motif and the resulting recombination event that replaces the endogenous *pfcss* gene with one mutated to encode Ser30. Both constructs included a HA-tag near the N-terminus of the PfCSS protein. In the grey box is the expected amino acid sequence expected after each insertion event. The HA-tagged *pfcss* gene that retained expression of C30 but inserted a HA-tag was successfully obtained and confirmed by sequencing (bottom panel). Parasites from multiple transfections with the construct that was identical to the former but would result in mutation of C30 to S30 was not successfully obtained. Therefore, we conclude that the C30 amino acid and the disulfide bond with PfPTRAMP was essential.

**Extended Data Fig. 4:**
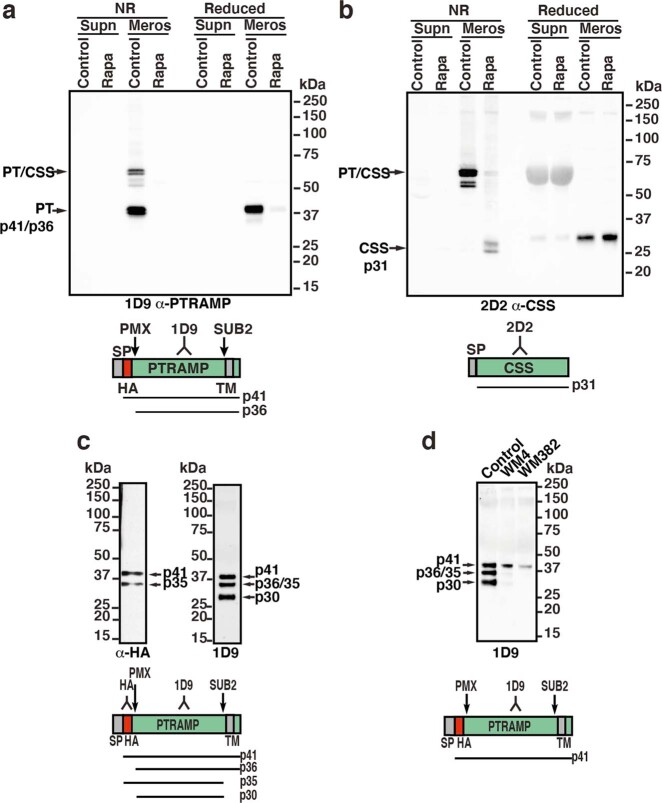
Conditional knockdown of PfPTRAMP and PfCSS confirms they form a heterodimer. **a and b**. Merozoites (Meros) and supernatants (Supn) probed with 1D9 monoclonal antibody to detect PfPTRAMP (**a**) and 2D2 monoclonal antibody to detect PfCSS (**b**) from 3D7 iKO PfPTRAMP and 3D7 iKO PfCSS in the absence (control) or presence of rapamycin (Rapa) and proteins run on SDS/PAGE under non-reducing (NR) or reducing (Reduced) conditions and an immuno-blot performed. Position of detected PTRAMP-CSS heterodimer and PfPTRAMP and PfCSS monomers are arrowed. Cartoons below panels show structure of PfPTRAMP and PfCSS, the position of the monoclonal epitopes and the processing due to PMX and SUB2. SP, Signal sequence. TM, transmembrane. **c**. Aspartic protease PMX processes PfTRAMP at the N-terminus. *P. falciparum* parasites that express a HA-tagged PfPTRAMP were probed with anti-HA or a specific monoclonal (1D9) to detect processed polypeptides. Molecular weight markers are shown in kDa on the left and predicted (p) sizes of the processed proteins on the right. Cartoon of PfPTRAMP is shown below the panels. Antibody symbol shows the position of the HA-tag (red) and the domain to which the monoclonal antibody 1D9 binds. Signal sequence SP and transmembrane TM (grey). SUB2 protease and the identified PMX cleavage site are shown with an arrow. Below the protein are the predicted molecular weights of each processed polypeptide. **d**. WM4 and WM382 inhibitors block processing of PfPTRAMP. PMX inhibitor WM4 used at 40 nM, dual PMX and PMIX inhibitor WM382, 2.5 nM. On the left are the predicted (p) molecular weights of each detected polypeptide. The molecular weight markers shown on the right in kDa. PfPTRAMP detected using the monoclonal antibody 1D9. A cartoon of the PfPTRAMP protein is shown below. An antibody symbol shows the position of 1D9 binding to the domain. Signal sequence SP and transmembrane TM (grey). SUB2 protease and the identified PMX cleavage site are shown with an arrow. Below the protein is predicted molecular weight due to WM4 and WM382 inhibition of PMX processing. Western blot in all panels were performed at least twice.

To determine whether PfPTRAMP and PfCSS form a complex, we first immunoprecipitated PfCSS–HA (hemagglutinin) resulting in enrichment of PfPTRAMP as detected by mass spectrometry (Extended Data Fig. 3). Second, immunoprecipitation of PfCSS–HA with anti-HA antibodies revealed a 65 kDa band under non-reducing conditions that migrated at 34 kDa (p34) when reduced (Fig. 4a). The reciprocal experiment with anti-PfPTRAMP monoclonal antibody (mAb) 1D9 detected the same 65 kDa band under non-reducing conditions and at 30–32 kDa when reduced. Third, conditional knockdown of PfPTRAMP expression disrupted the 65 kDa band so that the 33 kDa PfCSS protein was predominantly observed (Extended Data Fig. 4). Taken together, these data show PfPTRAMP and PfCSS form a disulfide linked heterodimer.

### PTRAMP–CSS bind Ripr and enhance PfRh5 erythrocyte binding

PfPTRAMP, PfCSS and PTRAMP–CSS heterodimer were used to test their ability to bind the RCR complex and human erythrocytes. Initially, we determined whether PfPTRAMP and PfCSS were proteolytically cleaved to ensure that the equivalent of the mature processed proteins was expressed. PfCSS was not processed; however, PfPTRAMP was cleaved by plasmepsin X (PMX) (Extended Data Fig. 4). PfCSS and PfPTRAMP constructs were designed, and the monomeric and PTRAMP–CSS dimer were expressed and purified to homogeneity (Fig. 4b).

Binding of PTRAMP–CSS and PfCSS to PfRipr was detected, with a moderate affinity of equilibrium dissociation constant (*K*_D_)3.6 ± 0.09 μM and *K*_D_ 1.1 ± 0.07 μM, respectively (Fig. 4c,d). The monomers of PfRh5 or CyRPA showed no binding to PTRAMP–CSS (Fig. 4e). The ability of PfCSS to bind to PfRipr indicated it bound PfRipr and that PfPTRAMP does not contribute to this interaction (Fig. 4c,d). Finally, PfPTRAMP bound to PfCSS at a lower affinity of *K*_D_ 19 ± 5 μM (Fig. 4f), suggesting that while the interacting surface between these two proteins is complementary, the disulfide bond is critical for formation and stability of the heterodimer. Our finding that the unpaired cysteine residue (C30) in PfCSS (see below), proposed to form the disulfide bonded heterodimer, was essential for growth supported the functional importance of PTRAMP–CSS (Extended Data Fig. 1).

**Extended Data Fig. 5:**
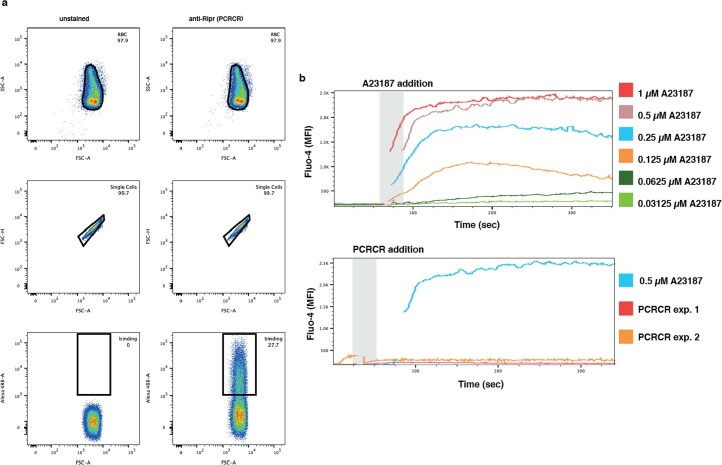
Gating strategy for detection of erythrocyte binding Ca^2+^ uptake by flow cytometry. **a**. Example scatter plots of the gating strategy for the erythrocyte binding assay showing unstained erythrocytes and erythrocytes incubated with PCRCR and detected with anti-PfRipr polyclonal sera and Alexa-488. The erythrocyte population was gated with SSC-A and FSC-A (top), then doublets were excluded using FSC-H and FSC-A (middle). For determining complex binding to erythrocytes, a cutoff of >10^3^ was used (bottom). Gating was performed in an identical manner for all other antibody and antigen combinations. **b**. Kinetic plot of A23187 stimulation of erythrocytes pre-loaded with Fluo-4 AM. A titration of A23187 shows the responsiveness of the Fluo-4 AM labelled erythrocytes to the calcium ionophore (top). The mean fluorescence intensity for the Fluo-4 AM signal is plotted. Kinetic plot of PCRCR addition to erythrocytes in comparison to 0.5 μM A23187 stimulation (bottom).

PfPTRAMP, PfCSS, PTRAMP–CSS and PTRAMP–CSS–Ripr were incubated with human erythrocytes, and no direct binding was detected (Fig. 4g and Extended Data Fig. 5a). However, when PTRAMP–CSS was added with the RCR complex, significant binding was detected. Consistent with our previous studies, PfRipr and CyRPA bound erythrocytes only as part of the tripartite RCR complex^11^. While PfRh5 bound erythrocytes either alone or in the RCR complex, it bound most efficiently in the pentameric complex with PCRCR (Fig. 4g). When added to human erythrocytes, PCRCR was not sufficient to induce a basal increase in Ca^2+^ in vitro as shown previously for RCR (Extended Data Fig. 5b)^11^. These results show PCRCR enhances the ability of PfRh5 to bind the receptor basigin on erythrocytes.

### PfCSS and PfPTRAMP nanobodies inhibit merozoite invasion

**Fig. 5:**
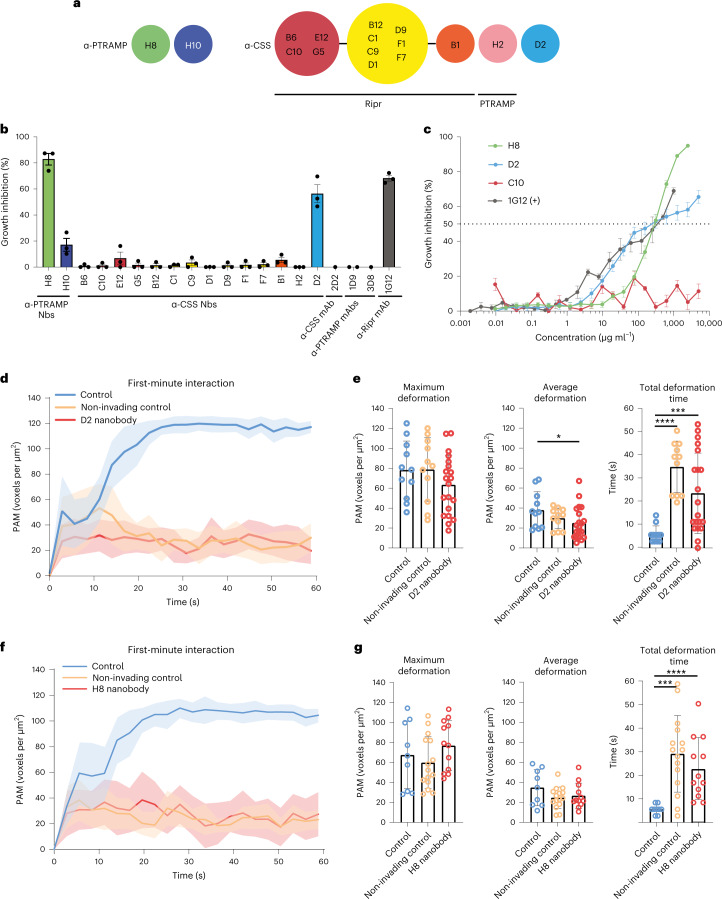
α-PfPTRAMP and α-PfCSS nanobodies inhibit merozoite invasion of erythrocytes. **a**, Epitope bins of α-PfPTRAMP and α-PfCSS nanobodies (Nbs). α-PfCSS nanobodies that compete with PfRipr and PfPTRAMP are indicated. See also Extended Data Fig. 6. **b**, Growth inhibition of parasites by α-PfPTRAMP and α-PfCSS nanobodies at 1 mg ml^−1^. *N* = 3; data are shown from three independent experiments, with data points representing the mean from one experiment, performed in triplicate. **c**, Growth inhibition dilution series for α-PfPTRAMP nanobody H8 and α-PfCSS nanobodies D2 and C10. α-PfRipr mAb 1G12 was used as a positive control^19^. Per cent growth inhibition is the mean of three independent experiments, performed in triplicate. The s.e.m. is shown. **d**, PAM time plot showing the first-minute interaction by invading control (blue, *n* = 11), non-invading control (orange, *n* = 11) and D2 nanobody-treated parasites (5 mg ml^−1^) (red, *n* = 20) with neighbouring erythrocytes after parasite egress, where *t* = 0 represents the timepoint immediately before interaction began. Solid lines represent mean values, and shaded regions represent ±95% CI. **e**, Maximum deformation, average deformation and total deformation time during the first-minute interaction by invading control (blue, *n* = 11), non-invading control (orange, *n* = 11) and D2 nanobody-treated (red, *n* = 20) parasites. Bar heights represent mean values, and error bars represent s.d. Mann–Whitney two-tailed test; *P* values left to right: *P = 0.0487, ****P < 0.0001, ****P = 0.0007. **f**, PAM time plot showing the first-minute interaction by invading control (blue, *n* = 10), non-invading control (orange, *n* = 15) and H8 nanobody-treated parasites (red, *n* = 12) (1.25 mg ml^−1^) with neighbouring erythrocytes after parasite egress, where *t* = 0 represents the timepoint immediately before interaction began. Solid lines represent mean values, and shaded regions represent ±95% CI. **g**, Maximum deformation, average deformation and total deformation time during the first-minute interaction by invading control (blue, *n* = 9), non-invading control (orange, *n* = 15) and H8 nanobody-treated (red, *n* = 12) parasites. Bar heights represent mean values, and error bars represent s.d. Mann–Whitney two-tailed test *P* values left to right: ****P < 0.0001, ***P = 0.0004. Source data

**Extended Data Fig. 6:**
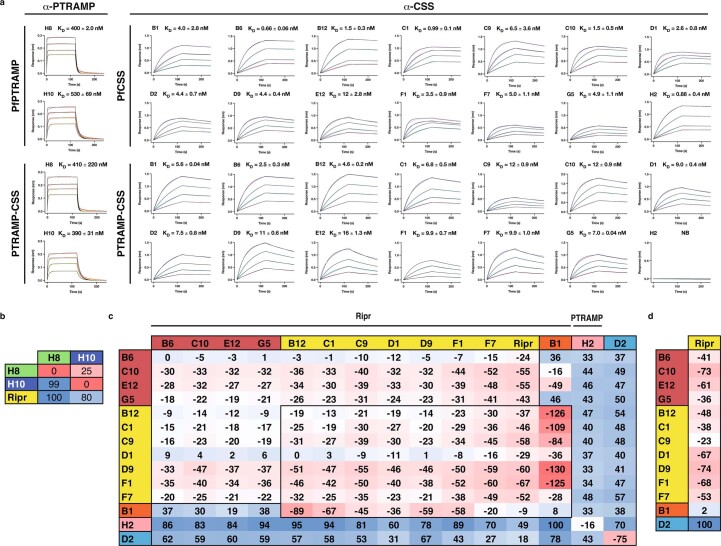
Characterization of α-PfPTRAMP and α-PfCSS nanobodies. **a**. Representative sensorgrams and 1:1 model best fit (black) for nanobody binding to PfTRAMP, PfCSS and PTRAMP-CSS, determined by biolayer interferometry. A 2-fold dilution series was used, starting at 2500 nM (brown), 1250 nM (red), 625 nM (orange), 313 nM (yellow) and 156 nM (wheat) for α-PfPTRAMP nanobodies and 250 nM (light pink), 125 nM (purple), 62.5 nM (cyan), 31.25 nM (teal) and 15.63 nM (pink) for α-PfCSS nanobodies. Epitope binning of α-PfPTRAMP nanobodies against **b**. PTRAMP-CSS and α-PfCSS nanobodies against **c**. PfCSS or **d**. PTRAMP-CSS. Primary nanobodies tested are listed in the left column, while secondary competing nanobodies are listed at the top. Data indicate the percent of competing nanobody or PfRipr binding compared to the maximum competing nanobody response. Boxes are coloured on a sliding scale, with red representing competition and blue representing non-competition. Nanobodies are coloured according to their epitope bins as in Fig. 5a.

We generated nanobodies to PfCSS and PfPTRAMP and determined the binding affinities, binding sites and ability to block binding to PfRipr (Fig. 5a, Extended Data Fig. 6, and Supplementary Tables 2 and 3). Anti-PfPTRAMP nanobodies H8 and H10 bound to distal sites on PTRAMP–CSS and did not block binding to PfRipr (Fig. 5a and Extended Data Fig. 6b). Anti-PfCSS nanobodies bound to three distinct sites on PfCSS. The first site comprised 12 out of the 14 nanobodies tested, and all competed with PfRipr for binding to PfCSS (Fig. 5a and Extended Data Fig. 6c). Within this bin, nanobodies bound to three overlapping epitopes. The second and third sites were distinct from the PfRipr binding site and comprised nanobodies H2 and D2. H2 competed with PfPTRAMP for binding to PfCSS. D2 nanobody bound to a site distal to the PfRipr and PfPTRAMP binding sites and did not block binding of PTRAMP–CSS to PfRipr (Extended Data Fig. 6d).

**Extended Data Fig. 7:**
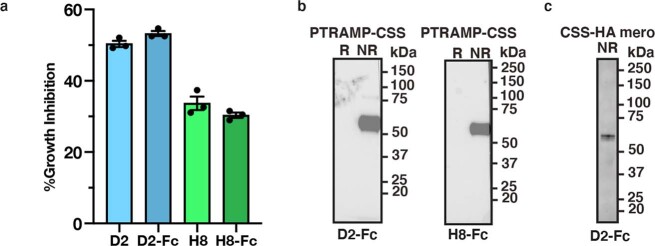
Nanobody-Fc fusion proteins specific to PfPTRAMP and PfCSS inhibit parasite growth and recognize PTRAMP-CSS in merozoites. **a**. Growth inhibition of parasites by D2 and H8 nanobodies and nanobody-Fc fusion proteins. Nanobodies and nanobody-Fc fusion proteins were tested at the EC_50_ concentration for growth inhibition of *P. falciparum*; D2 at 283 μg/ml and D2-Fc at 1.68 mg/mL or 21.2 μM; H8 at 288 μg/mL and H8-Fc at 1.72 mg/mL or 21.7 μM. Data is shown from one independent experiment, performed in triplicate. Error bars indicate standard error of the mean. **b**. Recombinant PTRAMP-CSS were probed with D2-Fc or H8-Fc fusion proteins under reduced (R) and non-reduced (NR) conditions. Western blot experiment has been performed at least twice. **c**. PfCSS-HA merozoites were probed with D2-Fc under non-reduced conditions to detect PTRAMP-CSS. Western blot experiment was performed once.

**Extended Data Fig. 8:**
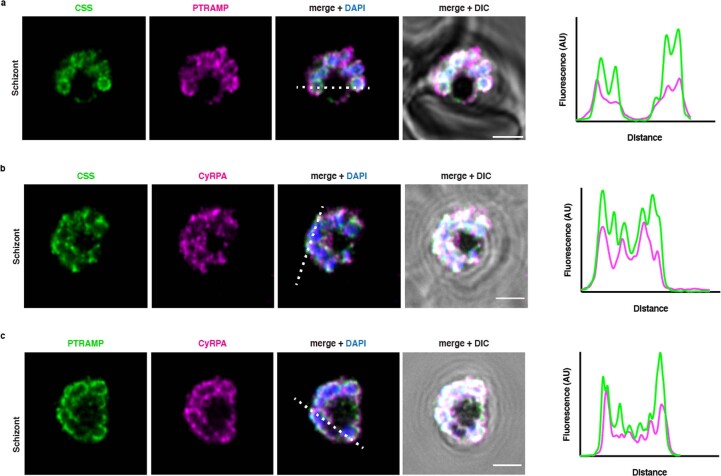
Co-localisation of PfCSS, PfPTRAMP and CyRPA in late schizonts of *P. falciparum* by immunofluorescence and super resolution microscopy. **a**. Localisation of PfCSS-HA (green) detected with anti-HA antibodies compared to PfPTRAMP detected using mAb 3D8 (purple). **b**. Localisation of PfCSS-HA (green) detected with anti-HA antibodies compared to CyRPA detected using mAb 8B9 (purple). **c**. Localisation of PfPTRAMP (green) detected with anti-HA antibodies compared to CyRPA detected using mAb 8B9 (purple). Shown for all panels is merge+DAPI where nuclei have been stained and PfCSS, PfPTRAMP and DAPI channels have been merged. Fourth panels on the right includes merge of all as well as DIC (differential interference contrast). The far-right panels indicate co-localisation of each protein across the broken white line of the merge+DAPI panels. The scale bar in merge + DIC panels is 2 μM and is relevant for all panels.

The anti-PfCSS nanobody D2 inhibited parasite growth with potency comparable with 1G12 anti-PfRipr mAb (Fig. 5b)^19^. PfPTRAMP nanobodies H8 and H10 showed inhibitory activity, with the former nanobody showing over 80% inhibition, whereas anti-CSS (2D2) and anti-PTRAMP (1D9, 3D8) mAbs did not inhibit growth. Inhibition of growth was dose dependent for D2 nanobody (anti-PfCSS) and H8 (anti-PfPTRAMP) with a half maximal effective concentration (EC_50_) of 283 µg ml^−1^ and 288 µg ml^−1^, respectively (Fig. 5c). D2 and H8 nanobody–Fc fusion proteins also inhibited growth to similar levels as the nanobodies and were used to show specificity to PTRAMP–CSS in merozoites (Extended Data Fig. 7). D2-Fc and H8-Fc recognized recombinant PTRAMP–CSS in non-reducing conditions, suggesting they bind conformational epitopes. In addition, D2-Fc recognized PTRAMP–CSS in merozoites. The ability of D2 and H8 nanobodies and nanobody–Fc fusions to inhibit growth showed PTRAMP–CSS was exposed on the merozoite surface and plays an essential role in invasion. Nanobodies blocking binding of PfCSS to PfPTRAMP and PfRipr did not inhibit growth suggesting the PTRAMP–CSS–Ripr complex was pre-formed in micronemes before exposure on the surface during invasion and consistent with co-localization of PfCSS and PfPTRAMP with CyRPA in mature schizonts (Extended Data Fig. 8).

LLSM was used to confirm D2 and H8 nanobodies inhibited merozoite invasion of erythrocytes (Fig. 5d–g and Supplementary Table 1)^16,20^. Using anti-PfCSS D2 nanobody, 23 merozoites were imaged interacting with erythrocytes, and of these 3 invaded. For H8 nanobody, 12 merozoites were imaged interacting with the erythrocyte membrane, and none invaded. Consequently, D2 and H8 nanobodies blocked merozoite invasion to ~87% and 100%, respectively, in accordance with growth inhibition assays (Fig. 5c).

Parental merozoites deformed the membrane, and 42–44% successfully invaded (Fig. 5d,e and Supplementary Table 1). For D2 and H8 nanobody-treated merozoites, the PAM plateaued as observed for merozoites lacking PfRh5, PfPTRAMP or PfCSS function (Fig. 2). Maximum and average deformation was the same for parental and D2 or H8 nanobody-treated merozoites indicating these activities were normal when PfCSS or PfPTRAMP function was inhibited (Fig. 5d,g). In contrast, total deformation time was significantly increased consistent with merozoites mediating rounds of deformation in repeated attempts to invade, as observed for those lacking PfRh5, PfPTRAMP or PfCSS function (Fig. 5d,g). Therefore, D2 and H8 nanobodies inhibit PfCSS and PfPTRAMP function, respectively, and block the function of PCRCR in invasion.

### Structure of PfCSS–nanobody complexes

**Fig. 6:**
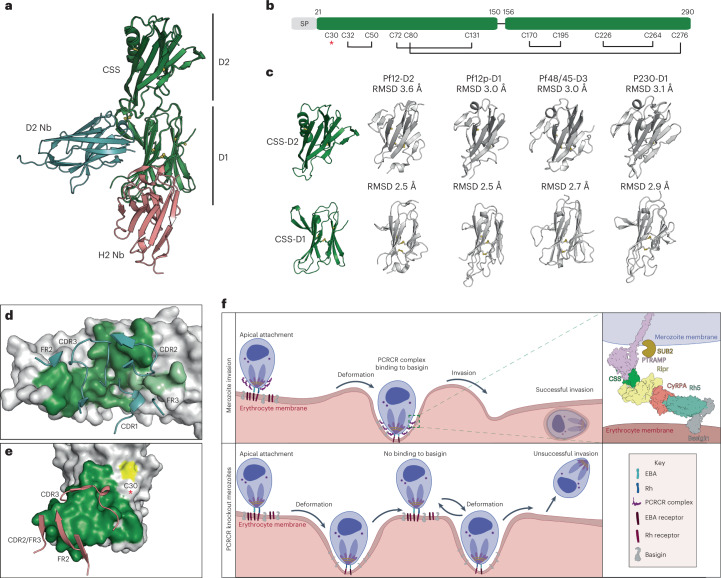
Anti-PfCSS nanobody recognition of the 6-Cys protein PfCSS. **a**, Crystal structures of D2 nanobody–PfCSS and H2 nanobody–PfCSS superimposed. **b**, Schematic representation of PfCSS showing disulfide bond pairing. The unpaired Cys30 is indicated by a red asterisk. **c**, Comparison of PfCSS D1 and D2 domains with previously determined crystal structures Pf12^52^, Pf12p^21^, Pfs48/45^53^ and Pfs230^54^. **d**, D2 nanobody CDRs and FRs interacting with PfCSS D1 (dark green) and PfCSS D2 (light green) domains. **e**, H2 CDRs and FRs interacting with PfCSS D1 domain near Cys30 (yellow) and highlighted by a red asterisk. **f**, Model depicting the role of the PCRCR complex in merozoite invasion. Top left: *P. falciparum* apical binding to human erythrocytes that occurs after initial interaction and membrane wrapping. Bottom: merozoites with either *pfptramp*, *pfcss*, *pfrh5* conditional knockouts, D2 or H8 nanobody. Top right: arrangement of the PCRCR complex binding to basigin on the erythrocyte membrane. SUB2 cleaves the ectodomain of PfPTRAMP.

**Extended Data Fig. 9:**
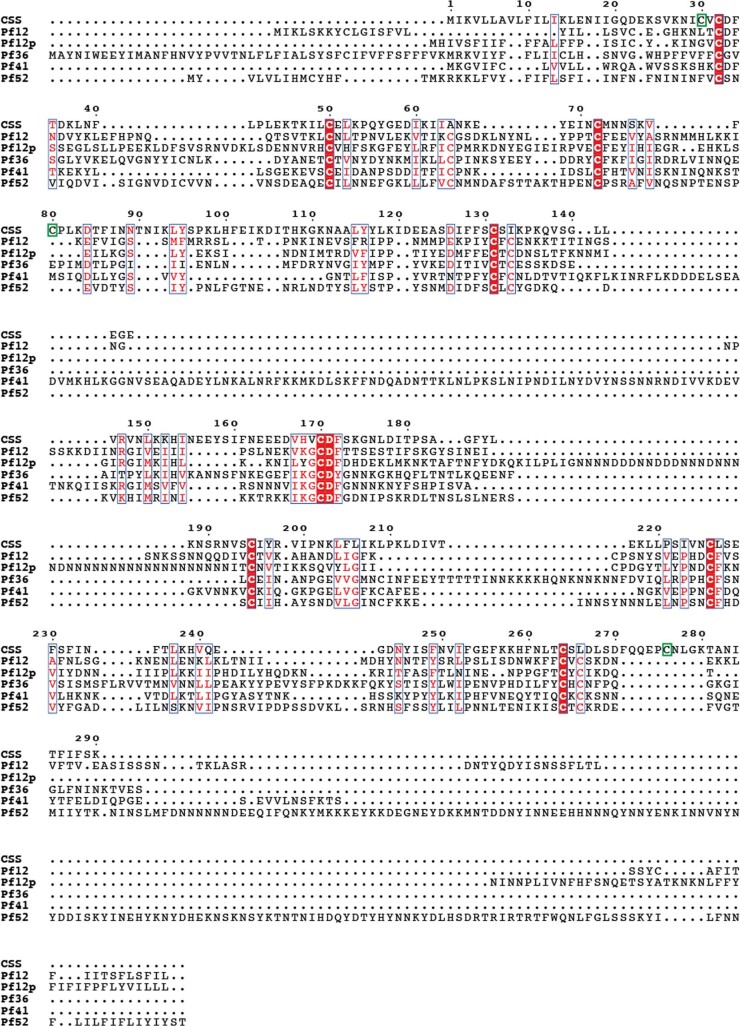
Amino acid sequence comparisons of the 6-cys family members including PfCSS, Pf12, Pf12p, Pf36, Pf41 and Pf52. Conserved cysteine residues are shown in dark red. The C30 cysteine in PfCSS that likely forms the disulfide bond with PfPTRAMP is marked in green. Less conserved residues are marked in light red.

Crystal structures of nanobodies D2 (inhibitory) and H2 (non-inhibitory) in complex with PfCSS were determined to a resolution of 4.13 Å and 2.00 Å, respectively (Supplementary Table 4). Analysis of the PfCSS sequence revealed similarity to the *Plasmodium* 6-Cys protein family, with 8 of the 11 cysteines conserved among five double domain *P. falciparum* 6-Cys proteins (Extended Data Fig. 9)^21,22^. Indeed, PfCSS adopts two ‘degenerate’ 6-Cys domains, denoted here as D1 and D2 (Fig. 6a,b). Both domains contain a β-sandwich fold with a mix of five on four parallel and antiparallel β-sheets. The D2 domain has an α-helix between residues 213 and 229 that replaces a β-sheet and loop present in other 6-Cys proteins (Fig. 6c)^21,22^. The eight conserved cysteines are paired to adopt the characteristic C1–C2 and C4–C5 6-Cys motifs in both D1 and D2 domains (Fig. 6a,b). An interdomain disulfide bond between residues C80 and C276 appears to rigidify the position of the two domains (root mean square deviation (rmsd) of PfCSS between two crystal structures, 0.49 Å). Importantly, C30 was solvent exposed and available for pairing to form a disulfide linkage with PfPTRAMP (Extended Data Fig. 3).

**Extended Data Fig. 10:**
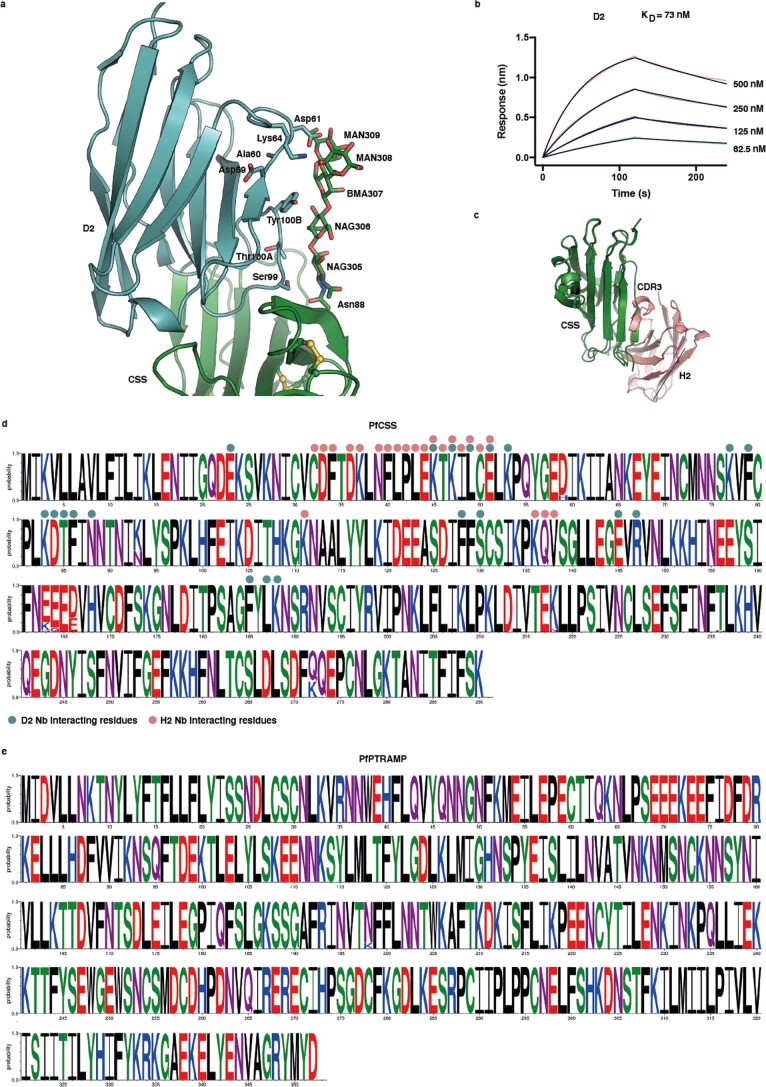
Nanobody recognition of PfCSS and sequence conservation of PfCSS and PfPTRAMP. **a**. D2 contacts an N-linked glycan on Asn88 of recombinant PfCSS. Interacting residues are shown as sticks. **b**. Representative sensorgram and 1:1 model best fit (black) for D2 binding to non-glycosylated PTRAMP-CSS determined by biolayer interferometry. A 2-fold dilution series was used, starting at 500 nM (light pink), 250 nM (lilac), 125 nM (purple), 62.5 nM (cyan). **c**. Superposition of the D1 domains from the D2-PfCSS and H2-PfCSS structures showing the β-strand is replaced by the H2 CDR3. **d**. Weblogo representation of PfCSS sequence diversity from 212 sequences from the PlasmoDB^55^. D2 and H2 interacting residues are denoted with teal and pink circles, respectively. **e**. Weblogo representation of PfPTRAMP sequence diversity from 214 sequences from the PlasmoDB.

Consistent with the competition binning data, D2 and H2 nanobodies bind to non-overlapping sites in PfCSS (Fig. 6a). D2 nanobody contacts both the D1 and D2 domains with most contacts targeted to one face of the β-sheet of the D1 domain (buried surface area (BSA) of 655 Å^2^) and the remaining to a loop in the D2 domain (BSA of 175 Å^2^) (Supplementary Table 5). The D2 CDR3 contributes more than half the total BSA (421 Å^2^), with CDR1, CDR2, FR2 and FR3 contributing the rest (Fig. 6d). CDR3 and FR3 of D2 form interactions with an N-linked glycan on Asn88 of PfCSS, with a BSA of 378 Å^2^ (Extended Data Fig. 10), which was glycosylated in the recombinant protein. Therefore, binding of D2 nanobody to a glycan deficient PTRAMP–CSS construct was determined using biolayer interferometry. Although affinity for the glycan deficient PTRAMP–CSS was tenfold lower, it showed notable binding (*K*_D_ = 73 nM compared with *K*_D_ = 7.5 nM), confirming D2 nanobody can bind to a ‘parasite-like’ PTRAMP–CSS heterodimer (Extended Data Fig. 10).

The non-inhibitory nanobody H2 interacts solely with the D1 domain of PfCSS (total BSA of 750 Å^2^) (Fig. 6e). It binds near the solvent-exposed Cys30, which we predict forms a disulfide bond with PfPTRAMP, consistent with the inability of H2 to bind PTRAMP–CSS. H2 binds in a side on orientation, with most contacts mediated by CDR3 (BSA 447 Å^2^); however, the CDR2, FR2 and FR3 all contribute (Fig. 6e and Supplementary Table 6). The CDR1 does not interact with PfCSS. Notably, the first six residues of PfCSS are missing from the crystal structure and are likely flexible. In the D2 nanobody–PfCSS crystal structure, these residues form the first β-strand of a β-sheet involved in the sandwich fold. However, in the H2–PfCSS crystal structure, this β-strand was replaced by the H2 CDR3, highlighting the flexibility of this β-strand in the absence of PfPTRAMP (Extended Data Fig. 10c).

The sequence diversity of D2, H2 and H8 nanobody epitopes in PfCSS and PfPTRAMP was analysed and found to be largely conserved (https://plasmodb.org/plasmo/app) (Extended Data Fig. 10). PfCSS has six polymorphisms, and PfPTRAMP has one polymorphism where the minor allele has a frequency of >5%. Notably, all PfCSS residues contacted by D2 and H2 nanobodies are conserved among all sequences (Extended Data Fig. 10). In addition, as PfPTRAMP had one polymorphic site, it is likely that H8 binds to a conserved epitope (Extended Data Fig. 10). The conserved nature of PfCSS and PfPTRAMP make them attractive targets for rational design of a vaccine eliciting strain-transcending antibodies that inhibit invasion.

## Discussion

The near isotropic data obtained from the high spatiotemporal resolution of LLSM imaging has provided a unique view of merozoite invasion providing a quantitative understanding of the surface contact between the pathogen and host cell^16^. This showed the PCRCR complex was responsible for capturing and anchoring the increased membrane surface contact formed between the merozoite and erythrocyte membranes created by strong deformations. This results in an irreversible interaction between the merozoite and erythrocyte and a stable platform for activation of the next steps for invasion and internalization of the merozoite into the erythrocyte.

PTRAMP–CSS was exposed on the surface of the invading merozoite and binds to the RCR complex, and we propose a model where it anchors the PCRCR complex to the parasite membrane through the transmembrane domain of PfPTRAMP^14^ to provide a platform for PfRh5 binding to basigin on the erythrocyte (Fig. 6f). PCRCR function was not required for initial interactions of the merozoite and binding at the apical tip that abuts the erythrocyte membrane after reorientation^12,23^. Nor was it required for weak or strong deformations of the erythrocyte membrane driven by the merozoite actomyosin motor^1,12^. The most likely mediators of the interaction at the merozoite tip are the EBA and PfRh protein families (excluding PfRh5)^2^. A consequence of these strong deformations would be an increase in surface area of the merozoite membrane in proximity with the membrane of the erythrocyte allowing the PCRCR complex to bind basigin across a broad area of the parasite, activating the insertion of PfRipr and PfRh5 into the erythrocyte membrane and providing an anchor on the parasite membrane^11^ (Fig. 6f). This would provide the ‘velcro’ that ties the membranes together, so the apical end of the merozoite remains embedded in the deformed host cell providing a stable and irreversible platform. When PCRCR function was inhibited, the interaction of the merozoite tip with the erythrocyte membrane and the strong deformations still occur; however, the lack of the ‘velcro’ to tie the parasite and host membrane together resulted in the merozoite bouncing back and proceeding through cycles of deformation until the energy driving the actomyosin motor would be depleted followed by parasite detachment (Fig. 6f)^1,9,24^.

Stabilization of merozoite–erythrocyte interactions would provide a base to establish the pore that allows Ca^2+^ entry into the erythrocyte^9,12,16^ and enable proteins to be injected under the erythrocyte membrane for formation of the moving junction by AMA1 and the RON complex^25,26^. However, while PCRCR function is required for these next steps, it is not directly involved. Once the moving junction has been established, it is propelled across the merozoite membrane towards the posterior of the parasite. The PCRCR complex would be released from the surface by processing of PfPTRAMP near the transmembrane domain by the protease SUB2 (Fig. 6f)^18^. This would free the PCRCR ‘velcro’ attachment between the parasite and host cell, allowing the moving junction to slide along the membranes to the posterior end for membrane sealing and completion of invasion and internalization.

Previously, it has been shown that the protein P113 binds to the N-terminus of PfRh5 and postulated that its glycosylphosphatidylinositol anchor bound the RCR complex to the merozoite membrane^27^. However, recent studies have shown that P113 function was not required for *P. falciparum* growth and is unlikely to be the membrane anchor for the RCR complex^28,29^.

PfCSS is a cryptic 6-Cys protein comprising two ‘degenerate’ 6-Cys domains, and this protein family typically mediates extracellular protein–protein interactions^30^, consistent with PfCSS binding to both PfPTRAMP and PfRipr. PTRAMP–CSS heterodimer formation would occur in the endoplasmic reticulum^31^ and then be trafficked to the micronemes where PfCSS could interact with PfRipr and CyRPA^9^ and form a tetrameric complex (PCRC). These proteins would then be exposed to PfRh5 at merozoite invasion as the micronemes empty their contents into the neck of the rhoptries allowing the PCRCR complex to form and spread onto the merozoite surface^9^. The inability of nanobodies that block binding of PfRipr to PfCSS and PfPTRAMP binding to PfCSS to inhibit merozoite invasion was consistent with formation of the PCRCR complex before exposure on the merozoite.

Identification of invasion inhibitory nanobodies to PfCSS and PfPTRAMP showed that these proteins have an essential role in the PCRCR complex and that they are exposed on the merozoite surface during invasion. The mechanism of D2 and H8 nanobody inhibition of PfCSS and PfPTRAMP remains to be determined, but it is possible that they either block insertion of PfRipr and PfRh5 into the erythrocyte membrane or inhibit three-dimensional changes in the PCRCR complex required for function. Indeed, the RCR complex binds with higher affinity to basigin, and the PCRCR complex shows even more efficient binding^11^. This would suggest that conformational changes to the PCRCR complex occur during interaction between basigin and PfRh5 that alter the affinity of binding to basigin. PfRh5 has a mobile structure, and formation of the RCR and PCRCR complex could lock in a conformation that binds more efficiently to basigin^11^.

The finding that nanobodies blocking Ripr–CSS or PTRAMP–CSS binding are non-neutralizing was consistent with these proteins associating before merozoite egress. Similar findings have been reported for CyRPA–Rh5 blocking antibodies^32–34^; however, a CyRPA–Rh5 blocking mAb was capable of inhibiting parasite growth which is somewhat at odds with this finding^19^. Previous studies have investigated whether polyclonal antibodies to PfCSS or PfPTRAMP could neutralize merozoite invasion; however, they showed no inhibitory activity^35,36^. An explanation, and consistent with our observations for the PfCSS and PfPTRAMP nanobodies, is that polyclonal responses are skewed to non-inhibitory immunodominant epitopes. Identification of invasion inhibitory epitopes on PfCSS and PfPTRAMP, both of which are highly conserved in *P. falciparum*, provides the molecular basis for rational design of immunogens.

## Methods

### Parasite, insect cell culture and antibodies

3D7 *P. falciparum* parasites were obtained from David Walliker, Edinburgh University. Asexual blood stage parasites were grown in in vitro culture as described^37^.

Sf21 insect cells were cultured in Insect-XPRESS protein-free with l-glutamine (Lonza, 10036636) medium at 28 °C. Expi293F cells were grown in Expi293 expression medium (ThermoFisher) at 37 °C, 8% CO_2_, 120 r.p.m.

In this study, we used: rat mAb, anti-HA (Roche 3F10, catalogue number 11867423001, lot 47877600); mouse mAbs, 1D9 and 3D8 anti-PfPTRAMP (this study), rat mAb 2D2 anti-PfCSS (this study), mouse mAbs 5B12, 7A6 and 8B9 anti-CyRPA^38^, 5A9 and 6H2 PfRh5^10^, mouse mAb 1G12 anti-Ripr^19^, rabbit anti-RON4 polyclonal^39^; rat pAb KM81 anti-PfCSS (this study); and rabbit pAb R1541 anti-Ripr^19^.

The mouse mAbs 1D9 and 3D8 that bound PfPTRAMP and the rat mAbs 2D2 mAb and pAb KM81 that bound PfCSS were made at the WEHI Antibody Facility as described in Supplementary Materials and Methods.

The following secondary antibodies labelled with Alexa 488/594 fluorophores (Life Technologies) and HRP antibodies were used: chicken anti-mouse 594 (catalogue number A21201, lot 42099 A), donkey anti-rat 488 (catalogue number A21208, lot 2310102), chicken anti-rabbit 594 (catalogue number A21442, lot 2110863), goat anti-mouse 488 (catalogue number A11001), goat anti-rabbit (catalogue number A11008). Peroxidase affinity pure goat anti-human IgG (H+L) (catalogue number 109-035-088, Jackson Immuno Research).

### Transgenic parasites and rhoptry and microneme secretion assay

Transgenic parasite lines were made using CRISPR–Cas9 with methods and oligonucleotides listed in Supplementary Materials and Methods^40^.

### Crosslinking, immunoprecipitation and mass spectrometry analysis

Parasites used for anti-HA antibody immunoprecipitation with and without cross-linking were synchronized and allowed to develop to schizonts; this is described in the Supplementary Materials and Methods.

### Live imaging with LLSM

A standard protocol was developed to ensure that parasites were at the same stages for each experiment. Two 30 ml dishes of asynchronous culture were synchronized with 5% sorbitol, as described^41^. In brief, the culture medium was removed, and the cells were incubated with five volumes of 5% sorbitol in a water bath at 37 °C for 8 min. The sorbitol was then washed off and fresh culture medium added back to the synchronized culture. This synchronization step was repeated 3 days after the first synchronization, and 10 nM rapamycin was added to one of the culture dishes after the second synchronization to induce *pfrh5* (3D7–Rh5iKO), *pfptramp* (3D7–PTRAMPiKO) and *pfcss* (3D7–CSSiKO) gene deletion in the relevant parasite lines. Two days after the second synchronization, late-stage parasites were isolated from the culture by magnet purification using LS columns attached to MACS MultiStand (Miltenyi Biotec).

Erythrocytes were resuspended at 0.5% haematocrit in RPMI-HEPES supplemented with 0.2% sodium bicarbonate and 5 mM sodium pyruvate (Gibco 11360070). To load uninfected erythrocytes with calcium indicator and stain the plasma membrane, the cells were incubated with 10 μM Fluo-4AM (Invitrogen F14201) for 1 h at 37 °C, and 1.5 μM Di-4-ANEPPDHQ (Invitrogen D36802) membrane marker was added for a further 1 h (refs. ^9,16^). The stained and loaded erythrocytes were washed three times and resuspended in phenol red free RPMI-HEPES supplemented with 5 mM sodium pyruvate, referred to as pyruvate medium hereafter^16^.

Purified schizonts were resuspended in culture medium and incubated with 10 nM Mitotracker Red CMXRos (Invitrogen M7512) for 30 min at 37 °C, 5% CO_2_. The stained schizonts were pelleted, and supernatant was removed before resuspending the schizonts in pyruvate medium. For sample mounting, an acid-washed 5 mm round glass coverslip (Warner Instruments CS-5R) was placed at the bottom of each well in an Ibidi eight-well plate (Ibidi 80826). Each well was then loaded with 200 μl pyruvate medium. Before imaging, 30 μl stained erythrocytes were loaded to a well and left to settle for at least 30 min. After that, 5–10 μl stained schizonts were added to the well and left to settle for around 15 min. A small amount of silicone gel was applied around the coverslip stage of the sample carrier, and a flat head tweezer was used to transfer the coverslip from the well to the sample carrier. The sample carrier was then attached to the microscope such that the coverslip was embedded in the microscope bath filled with 6–8 ml imaging medium that consisted of phenol red free RPMI-HEPES, 10% Albumax, 0.2% sodium bicarbonate, 5 mM sodium pyruvate, 0.25 mM CaCl_2_ and 10 μM Trolox (Santa Cruz 53188-07-1). Either 5 mg ml^−1^ D2 anti-CSS nanobody or 1.25 mg ml^−1^ H8 anti-PTRAMP nanobody was added to the imaging medium for invasion inhibition studies. The imaging experiments were performed on a custom-built LLSM microscope, constructed as outlined in as per licensed plans kindly provided by Janelia Farm Research campus^20^. Excitation light from either 488 nm or 589 nm diode lasers (MPB Communications) was focused to the back aperture of a 28.6 × 0.7 numerical aperture (NA) excitation objective (Special Optics) via an annular ring of 0.44 inner NA and 0.55 outer NA providing a light sheet with 10 μm length. Fluorescence emission was collected via a 25 × 1.1 NA water dipping objective (Nikon) and detected by either one or two scientific complementary metal–oxide–semiconductor cameras (Hamamatsu Orca Flash 4.0 v2). With the 488 nm excitation, emitted fluorescence was split using a 594 nm dichroic (Semrock) before passing through a LP 594 nm filter (Chroma) on camera A and 525/50 nm (Chroma) filter on camera B. This allowed simultaneous detection of Fluo-4 AM signals by camera B at 500–550 nm range and Di-4-ANEPPDHQ signals by camera A for wavelengths longer than 594 nm. With the 589 nm excitation, emitted fluorescence from Mitotracker Red CMXRos was detected on camera A with the same detection range as previous. All data were acquired in an imaging chamber (Okolabs) set to 36 °C and 5% humidified CO_2_.

For deconvolution, point spread functions were measured using 100 nm Tetraspeck beads on the surface of a 5 mm coverslip. Data were de-skewed and de-convolved using LLSpy, a Python interface for processing LLSM data. Deconvolution was performed using a Richardson–Lucy algorithm with 15 iterations with the point spread functions generated for each excitation wavelength.

### PAM plotting

Parasite–erythrocyte interactions were characterized by plotting the amount of surface contact at each timepoint for each event. The analysis was performed using Imaris (version 9.7.2, Bitplane) with Tracking module. A surface called ‘Erythrocytes’ was first created from the erythrocyte membrane channel with smoothing and absolute intensity setting. The threshold was adjusted either automatically or manually, on some occasions, to obtain an almost continuous surface on the erythrocyte of interest while maintaining the original boundary of the cell. Next, a surface called ‘All parasites’ was created from the parasite channel with smoothing and background subtraction setting. The threshold was adjusted accordingly to achieve reasonable values for parasite surface area (4–9 μm^2^), and 0.5 μm seed point value was used to split touching parasites. Next, a masked erythrocyte membrane channel was created from the erythrocyte surface by setting the voxel value inside the surface to 1 and outside the surface to 0. From the ‘All parasites’ surface, parasites that interact with the erythrocyte were then selected, by either automated tracking or manual selection, and duplicated into individual surfaces called ‘Parasite 1’, ‘Parasite 2’ and so on. For each parasite, all parts of the surface were selected and then unified and made into a single track. Finally, values of the ‘Intensity Sum’ from the masked erythrocyte membrane channel and the ‘Area’ at each timepoint were extracted from each parasite surface and exported to Microsoft Excel. The ‘Intensity Sum’ values represent the number of voxels in the erythrocyte membrane channel in contact with the parasite surface. The PAM values were then plotted from the Intensity Sum and normalized by the Area.

### *P. falciparum* schizont supernatant and merozoite preparations and analysis

Merozoite and supernatant preparations for SDS–PAGE and immunoblot analysis were performed as previously described^40^. Synchronized late trophozoite cultures were passed over LD magnetic columns (Miltenyi Biotech) to remove uninfected erythrocytes. Eluted parasites were adjusted to 5 × 10^6^ schizonts per ml and 150 μl added per well of a 96-well flat-bottomed culture dish. The assay dishes were further cultured for 16 h and a representative well smeared for Giemsa staining to ensure either that rupture had occurred normally (control well) or that rupture had been blocked when inhibitors were added. Parasites from each condition were spun at 10,000 × *g* for 10 min to collect the merozoite pellet and supernatant fractions. Proteins from both fractions were extracted with reducing sample buffer and separated on 4–12% or 3–8% acrylamide gel (NuPAGE, Invitrogen). When inhibitors WM4 and WM382 were at 40 nM and 2.5 nM final concentrations, respectively, a control dish without any protease inhibitor was also included. Parasites were eluted from columns with complete RPMI 1640 culture medium to which the appropriate inhibitor at the same concentration had been added.

### Expression and purification of PfCSS, PfPTRAMP, PTRAMP–CSS heterodimer, PfRipr, CyRPA and PfRh5

The gene for the PfPMX cleaved ectodomain of PfPTRAMP (residues 42 to 309) was subcloned into a modified pTRIEX2 vector with N-terminal SUMO and Flag tags followed by a Tobacco etch virus (TEV) protease cleavage site. One potential N-linked glycosylation site at Asn195 was removed by mutation of Thr197 to Ala. The construct was expressed in Sf21 insect cells and secreted into the medium as a soluble protein. The supernatant was purified by ANTI-FLAG M2 Affinity Gel (Merck) and size exclusion chromatography (S200 Increase 10/300 GL, Cytiva). Fractions containing PfPTRAMP were pooled and cleaved with TEV protease for 16 h at 4 °C. His-tagged TEV was removed via NiNTA agarose resin (Qiagen), and PfPTRAMP was further purified via another size exclusion chromatography (S200 Increase 10/300 GL, Cytiva). For biopanning anti-PfPTRAMP nanobodies and their kinetic characterization, a PfPTRAMP (42–309) construct with a C-terminal Avitag was generated and specifically biotinylated^42^. In addition, a PfPTRAMP construct comprising residues 25 to 309 with a C-terminal His-tag was used for bilayer interferometry binding studies to PfCSS; however, the purification was the same.

The gene for PfCSS (residues 20 to 290) was subcloned into a modified pTRIEX2 vector with a C-terminal Flag tag preceded by a TEV protease cleavage site. The construct was expressed in Sf21 insect cells and purified similarly to PfPTRAMP. The construct used for the alpaca immunization had no potential N-glycosylation sites mutated and was therefore glycosylated. The construct used in binding and crystallization studies had one glycan removed at Asn261, by mutation of Thr263 to Ala.

To generate disulfide-linked PTRAMP–CSS, PfPTRAMP (42–309) and PfCSS (20–290) constructs were co-expressed in Sf21 insect cells and secreted into the medium as a soluble protein. The supernatant was purified by ANTI-FLAG M2 Affinity Gel (Merck) and size exclusion chromatography (S200 Increase 10/300 GL, Cytiva). As both PfPTRAMP and PfCSS constructs contain a Flag tag, some free PfPTRAMP and PfCSS were present along with disulfide-linked PTRAMP–CSS after elution from the ANTI-FLAG M2 Affinity gel; however, they separated well from the disulfide-linked PTRAMP–CSS via size exclusion chromatography due to their differing sizes. Fractions containing PTRAMP–CSS were pooled and cleaved with TEV protease for 16 h at 4 °C. His-tagged TEV was removed via NiNTA agarose resin (Qiagen), and PTRAMP–CSS was further purified via another size exclusion chromatography (S200 Increase 10/300 GL, Cytiva). The purity of PTRAMP–CSS was assessed by SDS–PAGE and shown to be free from monomeric PTRAMP and CSS in non-reducing conditions (Fig. 4b). The PTRAMP–CSS construct used to test D2 nanobody glycan dependency and nanobody–Fc reactivity via western blot had four out of five potential N-linked glycan sites at Asn74, Asn192, Asn234 and Asn261 removed via mutation of the glycan site Thr or Ser to Ala. Mutation of the glycan at Asn283 led to no expression and so was not included. To test binding of nanobodies to PTRAMP–CSS, a biotinylated PTRAMP–CSS protein was generated using the PfPTRAMP (42–309) construct with a C-terminal Avitag.

The gene for PfRipr (residues 20 to 1086) was subcloned into pACGP67a with a C-terminal His tag. The construct was expressed in Sf21 cells and secreted into the medium as soluble protein. The supernatant was dialysed into 20 mM Tris pH 8, 150 mM NaCl. Imidazole was added to 10 mM final concentration, and PfRipr was purified by NiNTA agarose (Qiagen) and eluted in 20 mM Tris pH 8, 150 mM NaCl, 500 mM imidazole. The sample was further purified via size exclusion chromatography, using a S200 Increase 10/300 GL (Cytiva).

The gene for CyRPA (residues 29 to 362) was subcloned into a modified pcDNA3.4-TOPO plasmid with an N-terminal IL-2 signal sequence and a C-terminal Flag tag preceded by a TEV protease cleavage site. Three potential N-linked glycosylation sites at Asn145, Asn322 and Asn338 were removed by mutation of the glycan site Thr or Ser residues to Ala. The construct was expressed via transient transfection of Expi293F cells, and soluble protein was purified from the culture medium in a similar manner to PfPTRAMP described above.

The gene for PMX cleaved PfRh5 (residues 145 to 526) was subcloned into pACGP67a with a C-terminal C-tag. Three potential N-linked glycosylation sites as Asn214, Asn284 and Asn297 were removed by mutation of Thr or Ser residues to Ala. The construct was expressed in Sf21 cells and secreted into the medium as soluble protein. The supernatant was purified by CaptureSelect C-tagXL Affinity Matrix (ThermoFisher) and eluted with 20 mM Tris pH 7.5, 2 M MgCl_2_. The sample was further purified via size exclusion chromatography, using a S200 Increase 10/300 GL (Cytiva).

### Biolayer interferometry studies

Biolayer interferometry experiments were conducted at 25 °C to determine the affinity and epitope bins of selected proteins and nanobodies for PTRAMP–CSS, PfPTRAMP and PfCSS. For protein–protein binding kinetic studies, either PfRipr or PfPTRAMP was diluted into kinetics buffer (PBS, pH 7.4, 0.1% (w/v) BSA, 0.02% (v/v) Tween-20) at 20 μg ml^−1^ and immobilized onto Anti-Penta-His (His1K) biosensors (Sartorius). Following a 60 s baseline step, biosensors were dipped into wells containing twofold dilution series of either PTRAMP–CSS or PfCSS. Sensors were then dipped back into kinetics buffer to monitor the dissociation rate. For nanobody–PfCSS binding kinetic studies, nanobodies were diluted in kinetics buffer to 5 μg ml^−1^ and immobilized onto Ni-NTA (NTA) biosensors (Sartorius). Following a 60 s baseline step, biosensors were dipped into wells containing twofold dilution series of either PTRAMP–CSS or PfCSS. Sensors were then dipped back into kinetics buffer to monitor the dissociation rate. For nanobody–PfPTRAMP binding kinetic studies, biotinylated PfPTRAMP or PTRAMP–CSS were immobilized onto High Precision Streptavidin (SAX) biosensors (Sartorius). Following a 60 s baseline step, biosensors were dipped into wells containing twofold dilution series of anti-PfPTRAMP nanobodies.

For competition studies of the anti-PfCSS nanobodies, nanobodies were first diluted in kinetics buffer to 5 μg ml^−1^ and immobilized onto Ni-NTA (NTA) biosensors (Sartorius). Following a 30 s baseline step, biosensors were dipped into wells containing a negative control nanobody that does not bind the proteins under analysis to quench the sensors. Following another 30 s baseline step, biosensors were dipped into either PfCSS or PTRAMP–CSS. Following a final 30 s baseline step, biosensors were then dipped into a secondary nanobody or PfRipr to assess competition. Due to the moderate affinity of the anti-PfPTRAMP nanobodies, a premix format was employed. Nanobodies or PfRipr were first diluted to 10 μg ml^−1^ and immobilized onto Anti-Penta-His (His1K) biosensors. Following a 30 s baseline step, biosensors were dipped into wells containing a negative control nanobody that does not bind the proteins under analysis to quench the sensors. Following another 30 s baseline step, biosensors were then dipped into PTRAMP–CSS pre-incubated with a tenfold molar excess of competing secondary nanobody to assess competition.

Kinetics and competition data were analysed using Sartorius’ Data Analysis software 11.0. Kinetic curves were fitted to a 1:1 binding model. Mean kinetic constants reported are the result of two independent experiments. Data presented in Extended Data Fig. 6 represent the per cent of competing nanobody or PfRipr binding compared with the maximum competing nanobody response.

### Growth inhibition and flow cytometry of erythrocyte binding and Ca^2+^ flux

One-cycle growth inhibition and erythrocyte binding assays were performed as described previously^11,43^. The full methods are described in Supplementary Materials and Methods.

### Three-dimensional structure determination of PfCSS–nanobody complexes

For crystallization studies, PTRAMP–CSS and PfCSS alone were mixed with D2 and H2 nanobodies, respectively, in a 1:2 molar ratio, and excess nanobody was purified away via size exclusion chromatography (Superdex 200 Increase 10/300 GL, Cytiva). Complexes were then concentrated to 5 mg ml^−1^ and mixed 1:1 with mother liquor and set up in hanging or sitting drop crystallization experiments. D2 nanobody–CSS crystallized in 1.6 M ammonium sulfate, 0.1 M sodium chloride and 0.1 M sodium HEPES at pH 7.5 after 1 month and was cryoprotected in 15% (v/v) ethylene glycol. H2–PfCSS crystallized in 0.1 M bis-tris-propane pH 6.0, 17.5% (v/v) PEG3350, 0.2 M sodium malonate in 24 h and was cryoprotected in 15% (v/v) ethylene glycol. Data were collected at the MX2 beamline at the Australian Synchrotron, processed and merged using XDS^44^ and Aimless^45^. The positions of the H2 nanobodies in the H2–PfCSS crystal structure were first determined by molecular replacement using the structure of nanobody VHH-α204 from 5HVG with its CDR3 removed^46^. This solution was then used to build the two PfCSS molecules present in the asymmetric unit via AutoBuild^47^. This PfCSS structure was then used as a model for molecular replacement in the low-resolution crystal structure of D2 nanobody–CSS, along with VHH-72 from 6WAQ^48^. PfPTRAMP was not present in the D2–PTRAMP–CSS crystal structure. Presumably, PfPTRAMP and PfCSS dissociated during crystallization, and only D2 nanobody–PfCSS crystallized after 1 month in the high salt crystallization condition. Refinement of the structures was carried out using phenix.refine^49^ and iterations of refinement using Coot^50^.

### Reporting summary

Further information on research design is available in the Nature Research Reporting Summary linked to this article.

### Supplementary information

Supplementary Information Supplementary Tables 1–6, Videos 1 and 2 legends, Materials and Methods, and Source Data image for Fig. 1a–c.

Reporting Summary

Supplementary Video 1 Video of 3D7-PTRAMPiKO parasites grown without rapamycin and consequently displaying a normal invasion phenotype with PfPTRAMP, PfCSS and PfRh5 function. The human erythrocytes are loaded with Fluo4-AM allowing detection of Ca^2+^ (yellow) and the membrane stained with a membrane dye (purple). Parasites were stained with Mitotracker Red CMXRos (blue). Two merozoites interact with the membrane of the same erythrocyte and mediate deformation and a Ca^2+^ signal indicating a pore has been formed between the merozoite and erythrocyte membrane and this is followed quickly by successfully invasion, echinocytosis and entry into the erythrocyte.

Supplementary Video 2 Video of 3D7-PTRAMPiKO parasites grown with rapamycin and consequently lack PfPTRAMP function. The human erythrocytes are loaded with Fluo4-AM allowing detection of Ca^2+^ (yellow) and the membrane stained with a membrane dye (purple). Parasites were stained with Mitotracker Red CMXRos (blue). One merozoites in the middle of the erythrocyte interacts and shows clear rounds of deformation, however, successful invasion was not achieved and no Ca^2+^ (yellow) signal or echinocytosis was observed.

## Data Availability

The crystal structures reported in this manuscript have been deposited in the Protein Data Bank, www.rcsb.org (PDB ID codes 7UNY, 7UNZ). The mass spectrometry proteomics data have been deposited in the ProteomeXchange Consortium via the PRIDE^51^ partner repository with the dataset identifier PXD036746.

## Source data

Source Data Fig. 1a–c Unprocessed western blots.
Source Data Fig. 1d–f Statistical source data.
Source Data Fig. 2f–h Statistical source data.
Source Data Fig. 2f–h Statistical source data.
Source Data Fig. 4g Statistical source data.
Source Data Fig. 5b,c Statistical source data.
Source Extended Data Fig. 1g–i Statistical source data.
Source Extended Data Fig. 2a Statistical source data.
