# Supplementary material for: PCRCR complex is essential for invasion of human erythrocytes by Plasmodium falciparum
Source: Nat Microbiol. 2022 Nov 17;7(12):2039–53. doi: 10.1038/s41564-022-01261-2 (PMC9712106; doi:10.1038/s41564-022-01261-2)
Supplement: Supplementary file 1 — Supplementary Tables 1–6, Videos 1 and 2 legends, Materials and Methods, and Source Data image for Fig. 1a–c. [file 41564_2022_1261_MOESM1_ESM.pdf]

nature microbiology

Article https://doi.org/10.1038/s41564-022-01261-2

PCRCR complex is essential for invasion of human erythrocytes by *Plasmodium falciparum*

In the format provided by the authors and unedited

Supplementary Information for:

PCRCR complex is essential for invasion of human erythrocytes by *Plasmodium falciparum*

Stephen W. Scally[1,2], Tony Triglia[1], Cindy Evelyn[1], Benjamin A. Seager [1,2], Michal Pasternak [1,2], Pailene S. Lim [1,2], Julie Healer [1,2], Niall D. Geogheghan[1,2], Amy Adair[1], Wai-Hong Tham [1,2], Laura F. Dagley [1,2], Kelly L. Rogers [1,2], Alan F. Cowman [1,2,@]

[1]The Walter and Eliza Hall Institute of Medical Research, Parkville 3052, Australia

[2]University of Melbourne, Melbourne 3010, Australia

Correspondence to: cowman@wehi.edu.au

This file includes:

- Table S1 - S6
- Video S1 - S2 legends
- Supplementary Materials and Methods

Table S1. Number of merozoites interacting with erythrocytes visualised over time using Lattice Light Sheet Microscopy (LLSM) and observed invasion outcomes. *

Parasite and condition	Independent experiments (n)$	Total no. of merozoite observations	Successful Invasion	% Ca^{2+} flux &	Unsuccessful invasion	% Ca^{2+} flux&	% Successful invasion
3D7-Rh5iKO	4	21	9	100%	12	0	43%
3D7-Rh5iKO + Rapamycin	1	11	0	0	11	0	0
3D7-CSSiKO	2	21	11	100%	10	0	52%
3D7-CSSiKO + Rapamycin	3	15	0	0	15	0	0
3D7-PTRAMPiKO	3	21	12	0	9	0	57%
3D7-PTRAMPiKO + Rapamycin	4	15	0	0	15	0	0
3D7	5	19	8	100%	11	9.1%	42%
3D7 + D2 nanobody	4	23	3	100%	20	0	13%
3D7	3	27	12	83.3%	15	0	44%
3D7 + H8 nanobody	3	12	0	0	12	0	0

* Only merozoites that interact with the erythrocyte and deform the membrane were followed and quantitated.
$ Number of independent experiments to visualise the total number of merozoites indicated.
& % Ca^{2+} flux for those that interact with the erythrocyte.

Table S2. Anti-PfPTRAMP and -PfCSS nanobody sequences (CDR1, blue; CDR2, green; CDR3 maroon are coloured)

Nanobody	Antigen	Sequence
H8	PfPTRAMP	QVQLQESGGGLVQPGGSLRLSCAASGFTFSSYWMYWVRQAPGKGLEWVSAINTGGGSTAYADSVKGRFTISRDNAKNTLYLQMNSLKSDDTAVYYCVKARGSSWSDRVFDYW----GQGTQVTVSS
H10	PfPTRAMP	QVQLQESGGGLVQPGGSLRLSCAASGFTFDNYWMYWVRQAPGKGLEWVSAINTVGSHTYYADSVKGRFTISRDNAKNTLYLQMNSLKSEDTAVYYCAKGPWGFGTSGWGEMDYW--GKGTQVTVSS
B1	PfCSS	QVQLQESGGGLVQYGGSLRLSCAASGRTFSSYSMGWFRQAPGKEREFVAAISWTGGNTYYLGSTEGRFTISRDNAKNTVFLQMNNLKPEDTAVYYCAAKGRGSSWPTYDYR-----GQGTQVTVSS
B6	PfCSS	QVQLQESGGGLVQAGGSLRLSCAASGRTFSRYAMGWFRQAPGKEREFVAAISWSGGSTTYADSVKGRFTISRDNAKNTVYLQMNSLKPEDTAVYYCAAGDFIIGNMASEYEYW---GQGTQVTVSS
B12	PfCSS	QVQLQESGGGLVQAGGSLRLSCAASGRTFSRYAMGWFRQAPGNEREFVAAISWSGDSTYYGDSVKGRFTISRDNAXNTVYLQMNSLKPEDTAVYYCAAAGADVYMSTMRGEYDNW-GQGTQVTVSS
C1	PfCSS	QVQLQESGGGLVQAGGSLRLSCAASGRSFSSYAMGWFSQAPGKEREFVAAISWNGGSTYYADSVKGRFTISRNNAKNTVYLQMNSLKPEDTAVYYCAATEPEIYISTMAEGYDYW-GQGTQVTVSS
C9	PfCSS	QVQLQESGGGLVQAGDSLRLSCAASGRTFSSYAMGWFRQAPGKEREFVAAIAWNGGSTYYQDSVKGRFTISRDNAKNTVYLQMNSLKPEDTAVYYCAADDSYMSTMIHEYDYW---GQGTQVTVSS
C10	PfCSS	QVQLQESGGGLVQAGGSLRLSCVASGRTFSNDAMGWFRQAPGKEREFVAAATRNGLGTGYADSVKGRFTISRDNAKNTVYLQMNSLKPEDTAVYYCAAASDAYYSNQRAAYDYW--GQGTQVTVSS
D1	PfCSS	QVQLQESGGGLVQAGGSLRLSCAASGRTFSSLAMGWFRQPPGKEREFVAAISYSSGSTYYADSVKGRFTISRDNAKNTVYLQMNSLKPEDTAVYYCAVTPPDTYISTMGHEYDYW-GQGTQVTVSS
D2	PfCSS	QVQLQESGGGLVQAGGSLRLSCAASGRTFSSYAMGWFRQAPGKEREFVAAISYSGSNTYDADSVKGRFAISRDNAKNTVYLQMNSLKPEDTAVYYCAAAGVYSGTYTDTEFDYW--GQGTQVTVSS
D9	PfCSS	QVQLQESGGGLVQAGGSLTLSCAASGRTFSRSAMGWFRQAPGKGREFVAAISSGDTSTYYMDSVKGRFTISRDSSKNTVYLQMNNLRPEDTAVYYCAADPKSYITTMISEYDIW--GQGTQVTVSS
E12	PfCSS	QVQLQESGGGLVQAGGSLRLSCVASTRTFGNYAMAWFRQAPGKEREFVTTISASGGNTGAAPSVKGRFAISRDNAKNTVYLQMNSLKPEDTAVYYCAAASAYYSNDPARYTYW---GQGTQVTVSS
F1	PfCSS	QVQLQESGGGLVQAGGPLRLSCVASGRTFSLHLMGWFRQAPGKEREFVAAISGSGGSTYYADSVKGRFTISRDNAKNTVYLQMNSLKPEDTAVYYCAADLENYMSTMLDGYNYW--GQGTQVTVSS
F7	PfCSS	QVQLQESGGGSVQVGDSLSLSCVASGRTFSTYALGWFRQAPGKEREFVAAASANGKVAPVTGSLRDRFSISRDNAKNTMYLQMNNLKPEDTAVYYCAAATGSEVFISTSPNVYDIWGQGTQVTVSS
G5	PfCSS	QVQLQESGGGLVQAGGSLRLSCVASGRTFSSYGMGWFRQAPGKEREFVSAISSDAASRHYANSVKGRFTISRDNAKNTVTLQMSSLTPEDTAVYYCAAVHAYYSNYDGHYDYW---GQGTQVTVSS
H2	PfCSS	QVQLQESGGGLVQPGGSLRLSCAASGSIFSTNAMGWYRQAPGKQREQVATITSGSSTN-YADSVKGRFTISRDNAKNTVYLQMNSLKPEDTAVYYCNAAGATIDLADFGSW-----GQGTQVTVSS

Table S3. Binding kinetics of nanobodies to PfCSS, PfPTRAMP and PTRAMP-CSS.

	PTRAMP			PTRAMP-CSS			
**Nb**	**K_D (nM)**	**k_a (1/Ms)**	**k_d (1/s)**	**K_D (nM)**	**k_a (1/Ms)**	**k_d (1/s)**	**Fold Δ K_D**
**H8 ***	400 ± 2.00	$6.2 \times 10^{5} \pm 7.4 \times 10^{4}$	$2.5 \times 10^{-1} \pm 3.1 \times 10^{-2}$	410 ± 220	$7.9 \times 10^{5} \pm 3.5 \times 10^{5}$	$2.5 \times 10^{-1} \pm 2.6 \times 10^{-2}$	1.0
**H10 ***	530 ± 69	$1.7 \times 10^{5} \pm 1.9 \times 10^{4}$	$8.8 \times 10^{-2} \pm 1.7 \times 10^{-3}$	390 ± 31	$2.0 \times 10^{5} \pm 2.5 \times 10^{3}$	$7.7 \times 10^{-2} \pm 5.1 \times 10^{-3}$	0.74
	**CSS**			**PTRAMP-CSS**			
**Nb**	**K_D (nM)**	**k_a (1/Ms)**	**k_d (1/s)**	**K_D (nM)**	**k_a (1/Ms)**	**k_d (1/s)**	**Fold Δ K_D**
**B1** $^{\$}$	4.0 ± 2.8	$1.4 \times 10^{5} \pm 4.9 \times 10^{4}$	$3.9 \times 10^{-4} \pm 1.6 \times 10^{-4}$	5.6 ± 0.040	$1.6 \times 10^{5} \pm 1.5 \times 10^{3}$	$8.9 \times 10^{-4} \pm 1.0 \times 10^{-6}$	1.4
**B6** $^{\$}$	0.66 ± 0.06	$2.1 \times 10^{5} \pm 1.4 \times 10^{4}$	$1.4 \times 10^{-4} \pm 2.2 \times 10^{-5}$	2.5 ± 0.33	$2.1 \times 10^{5} \pm 1.9 \times 10^{4}$	$5.2 \times 10^{-4} \pm 2.1 \times 10^{-5}$	3.8
**B12** $^{\$}$	1.5 ± 0.31	$1.9 \times 10^{5} \pm 2.2 \times 10^{4}$	$2.9 \times 10^{-4} \pm 2.6 \times 10^{-5}$	4.6 ± 0.18	$1.8 \times 10^{5} \pm 1.6 \times 10^{4}$	$8.2 \times 10^{-4} \pm 4.3 \times 10^{-5}$	3.1
**C1** $^{\$}$	0.99 ± 0.11	$1.9 \times 10^{5} \pm 6.2 \times 10^{4}$	$3.0 \times 10^{-4} \pm 7.7 \times 10^{-5}$	6.8 ± 0.45	$1.6 \times 10^{5} \pm 6.5 \times 10^{3}$	$1.1 \times 10^{-3} \pm 1.2 \times 10^{-4}$	6.9
**C9** $^{\$}$	6.5 ± 3.6	$2.3 \times 10^{5} \pm 9.1 \times 10^{4}$	$1.1 \times 10^{-3} \pm 2.2 \times 10^{-4}$	12 ± 0.85	$2.2 \times 10^{5} \pm 1.0 \times 10^{4}$	$2.6 \times 10^{-3} \pm 3.1 \times 10^{-4}$	1.8
**C10** $^{\$}$	1.5 ± 0.46	$3.9 \times 10^{5} \pm 1.1 \times 10^{5}$	$5.6 \times 10^{-4} \pm 2.5 \times 10^{-5}$	4.8 ± 0.47	$2.6 \times 10^{5} \pm 3.2 \times 10^{4}$	$1.3 \times 10^{-3} \pm 3.0 \times 10^{-5}$	3.2
**D1** $^{\$}$	2.6 ± 0.78	$2.9 \times 10^{5} \pm 8.3 \times 10^{4}$	$6.8 \times 10^{-4} \pm 6.0 \times 10^{-6}$	9.0 ± 0.39	$2.1 \times 10^{5} \pm 1.4 \times 10^{4}$	$1.9 \times 10^{-3} \pm 3.5 \times 10^{-5}$	3.5
**D2** $^{\&}$	4.4 ± 0.70	$3.2 \times 10^{5} \pm 4.3 \times 10^{4}$	$1.4 \times 10^{-3} \pm 3.0 \times 10^{-5}$	7.5 ± 0.75	$1.3 \times 10^{5} \pm 5.0 \times 10^{3}$	$9.5 \times 10^{-4} \pm 5.6 \times 10^{-5}$	1.7
**D9** $^{\$}$	4.4 ± 0.40	$3.2 \times 10^{5} \pm 4.9 \times 10^{4}$	$1.3 \times 10^{-3} \pm 8.0 \times 10^{-5}$	11 ± 0.62	$2.5 \times 10^{5} \pm 2.4 \times 10^{4}$	$2.6 \times 10^{-3} \pm 4.0 \times 10^{-4}$	2.5
**E12** $^{\$}$	12 ± 2.8	$1.9 \times 10^{5} \pm 3.7 \times 10^{4}$	$2.1 \times 10^{-3} \pm 1.1 \times 10^{-4}$	16 ± 1.3	$2.3 \times 10^{5} \pm 1.5 \times 10^{4}$	$3.6 \times 10^{-3} \pm 5.5 \times 10^{-5}$	1.3
**F1** $^{\$}$	3.5 ± 0.87	$4.2 \times 10^{5} \pm 1.2 \times 10^{5}$	$1.4 \times 10^{-3} \pm 5.5 \times 10^{-5}$	9.9 ± 0.69	$3.2 \times 10^{5} \pm 1.5 \times 10^{4}$	$3.2 \times 10^{-3} \pm 8.0 \times 10^{-5}$	2.8
**F7** $^{\$}$	5.0 ± 1.1	$3.6 \times 10^{5} \pm 6.1 \times 10^{4}$	$1.8 \times 10^{-3} \pm 8.5 \times 10^{-5}$	9.9 ± 1.0	$2.4 \times 10^{5} \pm 7.5 \times 10^{4}$	$2.4 \times 10^{-3} \pm 1.8 \times 10^{-4}$	2.0
**G5** $^{\$}$	4.9 ± 1.1	$2.2 \times 10^{5} \pm 3.1 \times 10^{4}$	$1.1 \times 10^{-3} \pm 8.0 \times 10^{-5}$	7.0 ± 0.045	$2.6 \times 10^{5} \pm 1.3 \times 10^{4}$	$1.8 \times 10^{-3} \pm 9.5 \times 10^{-5}$	1.4
**H2** $^{@}$	0.88 ± 0.40	$1.8 \times 10^{5} \pm 3.0 \times 10^{4}$	$1.5 \times 10^{-4} \pm 4.6 \times 10^{-5}$	NB	-	-	-

* Anti-PfPTRAMP nanobodies H8 and H10 bind to distal sites on PTRAMP-CSS and did not block binding to PfRipr.

$^{\$}$ Anti-PfCSS nanobodies competed with PfRipr binding to PfCSS.

$^{@}$ H2 competed with PfPTRAMP for binding to PfCSS.

$^{\&}$ D2 bound to a site distal to the PfRipr and PfPTRAMP binding sites and did not block binding of the PTRAMP-CSS heterodimer to PfRipr.

Table S4. Data collection and refinement statistics.

	D2-PfCSS	H2-PfCSS
**Beamline**	MX2	MX2
**Wavelength (Å)**	0.953732	0.95366
**Space group**	$P3_221$	$P2_12_12$
**Cell dimensions**		
a,b,c **(Å)**	182.4, 182.4, 143.7	118.97, 190.4, 56.0
**α, β, γ (°)**	90, 90, 120	90, 90, 90
**Resolution (Å)**[a]	39.49-4.13 (4.46-4.13)	48.99-2.03 (2.07-2.03)
**No. molecules in ASU**	2	2
**No. observations**	244,435 (47,829)	1,126,541 (51,781)
**No. unique observations**	21,543 (4,391)	83,296 (4,251)
**Multiplicity**	11.3 (10.9)	13.5 (12.2)
**R_{merge} (%)**[b]	22.8 (153.3)	9.3 (146.0)
**R_{pim} (%)**[c]	7.1 (48.5)	2.7 (44.4)
**<I/σ I>**	7.7 (1.7)	14.0 (1.3)
**$CC_{½}$**	98.9 (61.5)	99.9 (72.2)
**Completeness (%)**	99.9 (100.0)	99.6 (93.2)
**Refinement Statistics**		
**Reflections (work)**	21,509	78,791
**Reflections (test)**	1,098	1,913
**Non-hydrogen atoms**	6,642	6,952
**Macromolecule**	6,328	6,166
**Water**	0	511
**Heteroatom**	314	275
**R_{work}[d] / R_{free}[e]**	21.6 / 25.1	18.3 / 21.6
**Rms deviations from ideality**		
**Bond lengths (Å)**	0.003	0.004
**Bond angle (°)**	0.49	0.63
**Ramachandran plot**		
**Favoured regions (%)**	93.1	98.0
**Allowed regions (%)**	6.9	2.0
**B-factors ($Å^2$)**		
**Wilson B-value**	147.3	35.0
**Average B-factors**	202.4	48.3
**Average macromolecule**	199.3	46.4
**Average heteroatom**	264.7	79.7
**Average water molecule**	-	49.2

[a] Values in parentheses refer to the highest resolution bin.

[b] $R_{merge} = \Sigma_{hkl} \Sigma_i \mid I_{hkl,i} - \langle I_{hkl} \rangle \mid / \Sigma_{hkl} \langle I_{hkl} \rangle$

[c] $R_{pim} = \Sigma_{hkl} [1/(N-1)]_{1/2} \Sigma_i \mid I_{hkl,i} - \langle I_{hkl} \rangle \mid / \Sigma_{hkl} \langle I_{hkl} \rangle$

[d] $R_{work} = (\Sigma \mid |F_o| - |F_c| \mid) / (\Sigma \mid |F_o|)$ - for all data except as indicated in footnote e.

[e] 5% of data were used for the R_{free} calculation

Table S5. Table of contacts between D2 and PfCSS.

CSS Residue (BSA Å^2)	Interaction Type	D2 Residue
**Thr20 (28.49)**		
Thr	VDW	Asp101, Trp103
**Gln21 (52.58)**		
Gln	VDW	Asp100D, Thr100E, Phe100G, Asp101, Trp103
**Glu23 (48.37)**		
Glu	VDW	Asp101, Tyr102
**Lys45 (32.90)**		
Lys	VDW	Asp100D, Thr100E
Lys$^{N\zeta}$	HB	Thr100E^{O}
**Lys47 (14.57)**		
Lys	VDW	Thr100E, Glu100F, Asp101
**Leu49 (9.03)**		
Leu	VDW	Tyr102
**Glu51 (15.07)**		
Glu	VDW	Arg27, Tyr32
**Lys53 (29.85)**		
Lys	VDW	Arg27
**Lys77 (39.65)**		
Lys	VDW	Val97, Ser99
Lys$^{N\zeta}$	HB/SB	Glu100F$^{O\varepsilon2}$
**Phe79 (11.27)**		
Phe	VDW	Tyr52A
**Lys83 (19.28)**		
Lys	VDW	Asn56, Tyr58
**Asp84 (92.21)**		
Asp	VDW	Tyr52A, Ser53, Gly54, Ser55, Asn56
Asp$^{O\delta2}$	HB	Ser52$^{O\gamma}$, Tyr52A^{N}, Ser53^{N}, Ser55$^{O\gamma}$
**Thr85 (70.76)**		
Thr	VDW	Asn56, Tyr58, Tyr98, Ser99, Gly100
Thr$^{O\gamma1}$	HB	Tyr58^{OH}
**Phe86 (60.04)**		
Phe	VDW	Tyr52A, Gly96, Val97, Tyr98, Ser99
Phe^{N}	HB	Tyr98^{O}
**Asn88 (15.04)**		
Asn	VDW	Ser99
**Phe128 (12.01)**		
Phe	VDW	Gly96
**Ser130 (9.46)**		
Ser	VDW	Val97
**Glu145 (13.93)**		
Glu	VDW	Val97
**Arg147 (80.77)**		
Arg	VDW	Tyr32, Gly96, Val97, Asp101, Asp101
Arg	HB/SB	Asp101$^{O\delta2}$
**Phe185 (55.86)**		

Phe	VDW	Ser31, Tyr52A
**Leu187 (90.38)**		
Leu	VDW	Ser30, Tyr52A, Ser53, Asn73
**Lys188 (28.23)**		
Lys	VDW	Ser53
**NAG305 (81.21)**		
NAG	VDW	Ser99, Gly100, Thr100A, Tyr100B
**NAG306 (112.25)**		
NAG	VDW	Ser99, Gly100, Thr100A, Tyr100B, Thr100C
**BMA307 (76.28)**		
BMA	VDW	Ala60, Asp61, Tyr100B
**MAN308 (80.91)**		
MAN	VDW	Asp59, Asp61, Lys64, Tyr100B
**MAN309 (27.74)**		
MAN	VDW	Asp61

Table S6. Table of contacts between H2 and PfCSS.

CSS Residue (BSA Å^2)	Interaction Type	H2 Residue
**Cys32 (2.18)**		
Cys	VDW	Leu100A
**Asp33 (11.71)**		
Asp	VDW	Leu100A
**Phe34 (7.52)**		
Phe	VDW	Leu100A
**Asp36 (15.53)**		
Asp	VDW	Trp103
**Lys37 (90.08)**		
Lys	VDW	Leu100A, Phe100D, Gly101, Ser102, Trp103
Lys$^{N\zeta}$	HB	Ser102$^{O\gamma}$
**Asn39 (56.74)**		
Asn	VDW	Arg45, Trp103
Asn$^{O\delta1}$	HB	Trp103$^{N\varepsilon1}$
**Phe40 (86.03)**		
Phe	VDW	Tyr37, Arg45, Glu46, Gln47, Phe100D, Trp103
**Leu41 (62.78)**		
Leu	VDW	Gln44, Arg45, Glu46, Gln47
Leu^{N}	HB	Arg45^{O}
Leu^{O}	HB	Gln47^{N}
**Pro42 (11.04)**		
Pro	VDW	Gln47
**Leu43 (135.09)**		
Leu	VDW	Gln47, Val48, Ala49, Thr50, Asn58, Tyr59, Ala60
Leu^{N}	HB	Gln47$^{O\varepsilon1}$
**Glu44 (18.30)**		
Glu	VDW	Asn58
Glu$^{O\varepsilon2}$	HB	Asn58$^{N\delta2}$
**Lys45 (18.35)**		
Lys	VDW	Gly96, Ala97
**Thr46 (39.97)**		
Thr	VDW	Gly96, Ala97, Ile99
Thr$^{O\gamma1}$	HB	Gly96^{O}
**Lys47 (44.63)**		
Lys	VDW	Ala97, Thr98, Ile99
Lys^{N}	HB	Ala97^{O}
Lys^{O}	HB	Ile99^{N}
**Ile48 (57.16)**		
Ile	VDW	Ile99, Asp100, Ile100A, Phe100D
**Leu49 (47.98)**		
Leu	VDW	Thr98, Ile99, Asp100, Ile100A
Leu^{N}	HB	Ile99^{O}
Leu^{O}	HB	Leu100A^{N}

**Cys50 (17.81)**		
Cys	VDW	Asp100, Ile100A
**Glu51 (1.02)**		
Glu		
**Lys109 (6.22)**		
Lys	VDW	Lys109
**Lys136 (1.96)**		
Lys	VDW	Gln44
**Gln137 (19.07)**		
Gln	VDW	Gln44
Gln^{O}	HB	$Gln^{N\varepsilon 2}$
**Val138 (6.36)**		
Val	VDW	Gln44

Video S1. Video of 3D7-PTRAMPiKO parasites grown **without** rapamycin and consequently displaying a normal invasion phenotype with PfPTRAMP, PfCSS and PfRh5 function. The human erythrocytes are loaded with Fluo4-AM allowing detection of Ca^{2+} (yellow) and the membrane stained with a membrane dye (purple). Parasites were stained with Mitotracker Red CMXRos (blue). Two merozoites interact with the membrane of the same erythrocyte and mediate deformation and a Ca^{2+} signal indicating a pore has been formed between the merozoite and erythrocyte membrane and this is followed quickly by successfully invasion, echinocytosis and entry into the erythrocyte.

Video S2. Video of 3D7-PTRAMPiKO parasites grown **with** rapamycin and consequently lack PfPTRAMP function. The human erythrocytes are loaded with Fluo4-AM allowing detection of Ca^{2+} (yellow) and the membrane stained with a membrane dye (purple). Parasites were stained with Mitotracker Red CMXRos (blue). One merozoites in the middle of the erythrocyte interacts and shows clear rounds of deformation, however, successful invasion was not achieved and no Ca^{2+} (yellow) signal or echinocytosis was observed.

Supplementary Materials and Methods

Parasite, insect cell culture and antibodies

3D7 *P. falciparum* parasites were obtained from Dr David Walliker, Edinburgh University. Asexual blood stage parasites were grown in *in vitro* culture [1] in O^{+} erythrocyte (Australian red-cross bloodbank, South Melbourne, Australia) at 4% hematocrit in Roswell Park Memorial Institute (RPMI) 1640 medium supplemented with 26 mM 4-(2-hydroxyethyl)piperazine-1-ethanesulfonic acid (HEPES), 50 µg/ml hypoxanthine, 20 µg/ml gentamicin, 0.2% $NaHCO_3$,

0.25% Albumax IITM (Gibco), and 5% heat-inactivated human serum. Cultures were incubated at 37°C in a mix of 94% N_2, 1% O_2 and 5% CO_2.

Sf21 insect cells were cultured in Insect-XPRESS Protein-free with L-Glutamine (Lonza, 10036636) medium at 28°C. Expi293F cells were grown in Expi293™ Expression medium (Thermofisher) at 37 °C, 8% CO_2, 120 RPM.

Antibodies and monoclonal antibodies were raised in rats and mice and all procedures approved by the Walter and Eliza Hall Institute of Medical Research Animal Ethics Committee. Animals were housed in open top cages with irritated feeding and autoclaved bedding. They were checked daily with a dark/light cycle of 12 hr, 7 PM-7 AM dark, 7 AM-7 PM light, temperature was set to 21 °C, ranging from 18-24 °C, and humidity was at approximately 40 % but not controlled. Immunization and handling of the alpaca for scientific purposes was approved by Agriculture Victoria, Wildlife and Small Institutions Animal Ethics Committee, project approval No. 26-17.

In this study, we used: rat mAb, anti-HA (Roche 3F10, Cat.: 11867423001, Lot: 47877600); mouse mAbs, 1D9 and 3D8 anti-PfPTRAMP (this study), rat mAb 2D2 anti-PfCSS (this study), mouse mAbs 5B12, 7A6 and 8B9 anti-CyRPA [2], 5A9 and 6H2 PfRh5 [3] , mouse mAb 1G12 anti-Ripr [4], rabbit anti-RON4 polyclonal [5]; rat pAb KM81 anti-PfCSS (this study); rabbit pAb R1541 anti-Ripr [4].

The mAbs 1D9 and 3D8 that bound to PfPTRAMP were raised in mice at the WEHI Antibody Facility, by immunising with recombinant PfPTRAMP expressed and purified from insect cells. Briefly, PfPTRAMP (N25-K309) between the end of the signal sequence and the start of the transmembrane domain was recodoned for insect cell expression (Genscript) and cloned into an insect cell expression vector bearing an N-terminal gp67 signal peptide, a SUMO tag, a FLAG tag and a tobacco etch virus (TEV) protease cleavage site.

The 2D2 mAb and pAb KM81 that bound to PfCSS were made in rats, at the WEHI Antibody Facility, by immunising with PfCSS recombinant protein expressed and purified from insect cells. PfCSS (Q21-K290) after the end of the signal sequence was recodoned for insect cell expression (Genscript) and cloned into an expression vector bearing an N-terminal gp67 signal peptide and a C-terminal fusion tag comprising a TEV site and a FLAG tag.

The following secondary Alexa 488/594 fluorophores (Life Technologies) were used: chicken anti-mouse 594 (Cat.: A21201, Lot: 42099A), donkey anti-rat 488 (Cat.: A21208, Lot: 2310102), chicken anti-rabbit 594 (Cat.: A21442, Lot: 2110863), goat anti-mouse 488 (Cat.: A11001), goat anti-rabbit (Cat.: A11008).

Parasite lines expressing HA-tagged proteins

Transgenic parasite lines were made using CRISPR-Cas9 as previously described (Favuzza et al, 2020). Guide oligos designed to induce a double-stranded break in the corresponding genomic positions were cloned using InFusion into pUF1-Cas9G: CyRPA: GTCACGACAAAGGCGAGACA; Ripr: CAAGGTCATGTAGCTGTCAA; and Rh5: GACAGATGATGAAACCGAAG (for C-terminal tagging). The strategy involved generation of a guide plasmid and a plasmid that replaces the endogenous target gene with a tagged version (the homology-directed repair or HDR plasmid). The HDR plasmids assembled in a modified p1.2 plasmid encoding WR99210 resistance, were made in either 3 steps, with 5' and 3' flanks (~500 bp upstream or downstream from the guide sequence) amplified from 3D7 genomic DNA and a codon-optimised target gene sequence (Genscript) cloned downstream of the 5' flank or in two steps, where the 5' flank was synthesized and fused to the codon-optimised gene sequence (in the case of CyRPA). Linearized HDR plasmid (50 µg) and circular guide plasmid (100 µg) were transfected into synchronised 3D7 schizonts suspended in 100 µl of P3 primary cell solution. Program FP158 with the Amaxa P3 primary cell 4D Nucleofector X Kit L (Lonza) was used. Parasites with an integrated drug-resistance cassette were selected and maintained on 2.5 nM WR99210.

Parasite lines with conditional gene knockouts

Transgenic parasite lines were made as above except plasmids were transfected into the Pfs47-DiCre line [6] to enable regulated deletion of specific genes using the dimerisable Cre system. Guide oligos for InFusion cloning were: PfRh5: GACAGATGATGAAACCGAAG; PTRAMP: TTTGTGTTCATGTAATTTGA; and PfCSS: ATTGGAAAATATCATAGGGC. The HDR plasmids were made as for the HA-tagged parasites, except the codon-optimised sequences included a loxP site within a *sera5* intron and a second loxP site, following the STOP codon, was part of the plasmid. For DiCre excision, synchronised schizont cultures were allowed to rupture till few ring stages were present, followed by sorbitol-synchronisation to remove schizonts, then grown with 10 nM rapamycin or DMSO.

Oligonucleotides

Oligonucleotides	Gene and name	Id number
GCGGCCGCCGTGCTATATAAACATATTTACG	Rh5 5' flank	TT683
TCAGATTTATCATCGATTTC	Rh5 5' flank	TT684
GAATTCGAAGATAGTATACAAGATAC	Rh5 3' flank	TT681
ACTAGTAATAAATAAAGAATATTCATTTGACAT	Rh5 3' flank	TT919
TAAGTATATAATATTgacagatgatgaaaccgaagGTTTTAGAGCTAGAA	Rh5 guide#	TT685
TTCTAGCTCTAAAACcttcggtttcatcatctgtcAATATTATATACTTA	Rh5 guide#	TT686
GCGGCCGCTTTTTGTATCTTACAGCTGCTCC	Ripr 5' flank	TT934
CATTCACCGCGGGATGATCTATAATAATGTTC	Ripr 5' flank	TT935
GAATTCAAATGTGTTTTAGAAGATAAATGTG	Ripr 3' flank	TT936
ACTAGTTACATGTTTGATGATCTACTTGG	Ripr 3' flank	TT937
TAAGTATATAATATTcaaggtcatgtagctgtcaaGTTTTAGAGCTAGAA	Ripr guide#	TT938
TTCTAGCTCTAAAACttgacagctacatgaccttgAATATTATATACTTA	Ripr guide#	TT939
GAATTCACATGGGGGTAAAA	CyRPA 3' flank	JV3
ACTAGTTATCCTTGCAGTAACCCCTTTTGT	CyRPA 3' flank	TT791
TAAGTATATAATATTgtcacgacaaaggcgagacaGTTTTAGAGCTAGAA	CyRPA guide#	JV1
TTCTAGCTCTAAAACtgtctcgcctttgtcgtgacAATATTATATACTTA	CyRPA guide#	JV2
GCGGCCGCTAAAATGCTCATTATAAAAACATGA	PTRAMP 5' flank	TT1127
CTCGAGATGTATAGAAATAATAAAAATGTA	PTRAMP 5' flank	TT1128
GAATTCAAATAATTGGGAACATTTTTTACA	PTRAMP 3' flank	TT1129
CTGCAGGTCTTTTGTAAAAGCTTTCCATGT	PTRAMP 3' flank	TT1130
TAAGTATATAATATTtttgtgttcatgtaatttgaGTTTTAGAGCTAGAA	PTRAMP guide#	TT1134
TTCTAGCTCTAAAACtcaaattacatgaacacaaaAATATTATATACTTA	PTRAMP guide#	TT1135
GCGGCCGCTCTCTTATATATTTTATTTCATATCA	CSS 5' flank	TT1123
CTCGAGTTTAATTAATATAAAAAGCACCG	CSS 5' flank	TT1124
GAATTCGAAAAGTGTAAAAAATATATGTG	CSS 3' flank	TT1125
CTGCAGAAAAATAATTTATTTGGGATAACCCT	CSS 3' flank	TT1126
TAAGTATATAATATTattggaaaatatcatagggcGTTTTAGAGCTAGAA	CSS guide#	TT1132

TTCTAGCTCTAAAACgccctatgatattttccaatAATATTATATACTTA	CSS guide#	TT1133

Guide oligos are 50 mers, where the 20 bases of the guide sequence that is present in the *P. falciparum* gDNA, is shown in lower case. The flanking 15 bases on either end, are required for the InFusion reaction to clone the guide into the pUF-cas9G plasmid.

Parasite growth assay

Transgenic ring stage parasites, in which a specific gene could be conditionally deleted, was synchronised at approximately 0.5 and 0.8% parasitaemia and grown in the presence of rapamycin or DMSO. Parasite smears were taken for Giemsa staining at approximately 22 hr, 29 hr, 46 hr, 52 hr, 70 hr, 76 hr and 94 hr post-invasion. One thousand cells were counted at each time point to determine the parasitaemia.

Rhoptry and microneme ligand secretion assay

The PfPTRAMP iKO parasite was used to analyse proteins in merozoites and supernatants as previously described (Protease Inhibition Assay)[7]. Synchronized late trophozoite/early schizont cultures to which protease inhibitors (WM4 or WM382) or Rapamycin had already been added, were passed over LS magnetic columns (Miltenyi Biotech) to remove uninfected erythrocytes. The PMX inhibitor WM4 was used at 40 nM, while the dual PMX and PMIX inhibitor WM382, was used at a 2.5 nM final concentration. For conditional gene deletion rapamycin was added (10 nM) to induce excision of the PfPTRAMP gene. A control dish without drug was also analysed. Parasites were eluted from columns with complete RPMI 1640 culture medium to which the appropriate inhibitor at the same concentration had been added. Eluted parasites were adjusted to $5x10^6$ schizonts/mL and 150 µl added per well of a 96-well flat-bottomed culture dish. The assay dishes were further cultured for 16 hr and a representative well from each condition smeared for Giemsa staining, to ensure either that rupture had occurred normally (control well) or that rupture had been blocked (WM4, WM382 and rapamycin conditions). Parasites from each condition were centrifuged at 10000 g/10 min to separate merozoite and supernatant fractions. Proteins were extracted with either non-reducing or reducing sample buffer and separated on 4%–12% or 3%–8% acrylamide gels (NuPAGE, Invitrogen).

For the HA-tagged PfCSS parasite, synchronised schizont cultures at ~5% parasitaemia, were allowed to rupture. The following day, cultures were centrifuged to remove cellular material, then the supernatant centrifuged at 10,000 g/20 min. Secreted proteins in the centrifuged

supernatant were extracted with either non-reducing or reducing sample buffer followed by SDS-PAGE.

SDS-PAGE and immunoblotting

HA-tagged parasites were synchronised and grown to schizont stage followed by saponin-lysis to remove uninfected erythrocytes. To activate conditional gene knockout ring stage parasites were grown in rapamycin or DMSO at the ring stage and analysed at schizont stages. Schizont pellets were lysed in reducing SDS sample buffer followed by analysis using precast Bis Tris NuPAGE polyacrylamide gels followed by transfer to nitrocellulose membranes by electroblotting. Blots were probed with HRP-conjugated anti-HA antibody (Roche) 1:1000 or for two-step methods, a primary antibody was followed by HRP-conjugated secondary antibody (Millipore). Bands were detected using ECL Plus Western blotting reagent (GE Healthcare) and the ChemiDoc Imaging System (Biorad).

Crosslinking and Immunoprecipitation

Parasites used for anti-HA antibody immunoprecipitation with and without cross-linking, were synchronised, allowed to develop to schizonts, followed by saponin lysis to remove uninfected erythrocytes and solubilised in 1% Triton X-100. In Fig. S1 A, 3D7-CyRPA-HA were the test parasites and as control 3D7 parasites were used with no DSP in both cases. For crosslinking protein complexes, 2 mM (final) of the thiol-cleavable crosslinker dithiobis(succinimidyl propionate) (DSP), was added to saponin-lysed pellets for 30 min at room temperature. In Fig. S1 D 3D7-CyRPA-HA parasites were used +DSP and 3D7 parasites +DSP were the control. In Fig. S1 B and E, 3D7-Ripr-HA parasites were used and 3D7 parasites + DSP was the control. In Fig. S1 C and F 3D7-Rh5-HA parasites were used and 3D7 parasites + DSP was the control. In all cases proteins were immunoprecipitated with HA beads. The reaction was quenched with 20 mM Tris, before proteins were extracted with 9 pellet volumes of TNET (1% TX100, 150 mM NaCl, 10 mM EDTA, 50 mM Tris, pH7.4). Protein extracts were incubated with agarose-bound anti-HA antibodies (Roche) and immunoprecipitants eluted with hot 0.5% SDS at 56°C for 5 min.

Trypsin digestion of HA immunoprecipitations

Eluates of HA-captured proteins derived from each biological replicate were prepared for mass spectrometry analysis using the FASP (filter aided sample preparation) method [8], with the

following modifications. Proteins were reduced with 10 mM Tris-(2-carboxyethyl)phosphine (TCEP), alkylated with 50 mM iodoacetamide, then digested with 1 µg sequence-grade modified trypsin gold (Promega) in 50 mM NH_4HCO_3 and incubated overnight at 37°C. Peptides were eluted with 50 mM NH_4HCO_3 in two 40 µl sequential washes and acidified in 1% formic acid (FA, final concentration).

Mass spectrometry analysis

The extracted peptide solutions from immunoprecipitation experiments were acidified (0.1% formic acid) and concentrated by centrifugal lyophilisation using a SpeedVac AES 1010 (Savant). For the HA-tagged PfCSS samples, peptides were reconstituted in 80 µl 2% ACN/0.1% FA and 3 µl separated by reverse-phase chromatography on a C18 fused silica column (inner diameter 75 µm, OD 360 µm × 25 cm length, 1.6 µm C18 beads) packed into an emitter tip (IonOpticks, Australia), using a nano-flow HPLC (M-class, Waters). The HPLC was coupled to a timsTOF Pro (Bruker) equipped with a CaptiveSpray source. Peptides were loaded directly onto the column at a constant flow rate of 400 nl/min with buffer A (99.9% Milli-Q water, 0.1% FA) and eluted with a 90-min linear gradient from 2 to 34% buffer B (99.9% ACN, 0.1% FA). The timsTOF Pro was operated in PASEF mode using Compass Hystar 5.1. Settings for the 11 samples per day method were as follows: Mass Range 100 to 1700m/z, 1/K0 Start 0.6 V·s/cm2 End 1.6 V·s/cm2, Ramp time 110.1ms, Lock Duty Cycle to 100%, Capillary Voltage 1600V, Dry Gas 3 l/min, Dry Temp 180°C, PASEF settings: 10 MS/MS scans (total cycle time 1.27sec), charge range 0-5, active exclusion for 0.4 min, Scheduling Target intensity 10000, Intensity threshold 2500, CID collision energy 42eV.

For the HA-tagged CyRPA, PfRipr, and PfRh5 samples, peptides were reconstituted in 80 µl 2% ACN/0.1% FA and 2 µl subjected to nanoflow reversed-phase liquid chromatography tandem mass spectrometry (LCMS/MS) on an Easy-nLC 1000 system (Thermo Fisher Scientific) coupled to a Q-Exactive HF (QE-HF) mass spectrometer equipped with a nanoelectrospray ion source and in-source column heater (Sonation) at 40 °C for automated MS/MS (Thermo Fisher Scientific). Peptide mixtures were loaded in buffer A (0.1% formic acid, 2% acetonitrile, Milli-Q water), and separated by reverse-phase chromatography using C_{18} fused silica column (packed emitter, internal diameter 75 µm, outer diameter 360 µm × 25 cm length, IonOpticks, Australia) using flow rates and data-dependent methods as previously described [9].

For the HA-tagged PfCSS samples, raw files consisting of high-resolution tandem mass spectrometry spectra were processed with MaxQuant (version 1.6.17) for feature detection and protein identification using the Andromeda search engine [10]. Extracted peak lists were searched against the *P. falciparum* 3D7 database and a separate reverse decoy database to empirically assess the FDR using a strict trypsin specificity allowing up to two missed cleavages. The minimum required peptide length was set to seven amino acids. The modifications included: carbamidomethylation of Cys was set as a fixed modification, whereas *N*-acetylation of proteins, the oxidation of Met was set as variable modifications. The 'match between runs' option in MaxQuant was used to transfer the identifications made between runs based on matching precursors with high mass accuracy. LFQ quantification was selected, with a minimum ratio count of 2. Peptide-spectrum match (PSM) and protein identifications were filtered using a target-decoy approach at an FDR of 1%. In the main search, precursor mass tolerance was 0.006 Da and fragment mass tolerance was 40 ppm. For the HA-tagged CyRPA, PfRipr, and PfRh5 samples, raw files were processed with MaxQuant (version 1.5.8.3) as described above, with the following differences: The mass tolerance for precursor ions and fragment ions was 20 p.p.m. and 0.5 Da, respectively.

Live-imaging with lattice light-sheet microscopy

A detailed standard protocol was developed to ensure parasites were at the same stages for each experiment. Two 30 mL dishes of asynchronous culture were synchronized with 5% sorbitol, as described [11]. In brief, the culture medium was removed, and the cells were incubated with 5 volume of 5% sorbitol in a water bath at 37°C for 8 min. The sorbitol was then washed-off and fresh culture medium added back to the synchronized culture. This synchronization step was repeated three days after the first synchronization and 10 nM of rapamycin added to one of the culture dishes after the second synchronization to induce *pfrh5* (3D7-Rh5iKO), *ptramp* (3D7-PTRAMPiKO) and *css* (3D7-CSSiKO) gene deletion in the relevant parasite lines. Two days after the second synchronization, late-stage parasites were isolated from the culture by magnet purification using LS columns attached to MACS MultiStand (Miltenyi Biotec).

Erythrocytes were resuspended at 0.5% hematocrit in RPMI-HEPES supplemented with 0.2% sodium bicarbonate and 5 mM sodium pyruvate (Gibco 11360070). To load uninfected erythrocytes with calcium indicator and stain the plasma membrane the cells were incubated with 10 μM Fluo-4AM (Invitrogen F14201) for 1 hr at 37°C and 1.5 μM Di-4-ANEPPDHQ (Invitrogen D36802) membrane marker was added for a further 1 hr [12,13]. The stained and loaded

erythrocytes were washed three times and resuspended in phenol red free RPMI-HEPES supplemented with 5 mM sodium pyruvate, referred as pyruvate medium hereafter [13].

Purified schizonts were resuspended in culture medium and incubated with 10 nM Mitotracker Red CMXRos (Invitrogen M7512) for 30 min at 37°C, 5% CO_2. The stained schizonts were pelleted and supernatant removed before resuspending the schizonts in pyruvate medium. For sample mounting, an acid-washed 5 mm round glass coverslip (Warner Instruments CS-5R) was placed at the bottom of each well in an Ibidi 8-well plate (Ibidi 80826). Each well was then loaded with 200 µL of pyruvate medium. Before imaging, 30 µL of stained erythrocytes were loaded to a well and left to settle for at least 30 min. After that, 5-10 µL of stained schizonts were added to the well and left to settle for around 15 min. A small amount of silicone gel was applied around the coverslip stage of the sample carrier and a flat head tweezer was used to transfer the coverslip from the well to the sample carrier. The sample carrier was then attached to the microscope such that the coverslip was embedded in the microscope bath filled with 6-8 mL of imaging medium consisted of phenol red free RPMI-HEPES, 10% Albumax, 0.2% sodium bicarbonate, 5 mM sodium pyruvate, 0.25 mM $CaCl_2$, and 10 µM Trolox (Santa Cruz 53188-07-1). Either 5 mg/mL of D2 anti-CSS nanobody or 1.25 mg/ml of H8 anti-PTRAMP nanobody was added to the imaging medium for invasion inhibition studies. The imaging experiments were performed on a custom-built LLSM microscope, constructed as outlined in as per licensed plans kindly provided by Janelia Farm Research campus [14]. Excitation light from either 488 nm or 589 nm diode lasers (MPB Communications) was focused to the back aperture of a 28.6x 0.7 NA excitation objective (Special Optics) via an annular ring of 0.44 inner NA and 0.55 outer NA providing a light sheet with 10 µm length. Fluorescence emission was collected via a 25x 1.1 NA water dipping objective (Nikon) and detected by either one or two sCMOS cameras (Hamamatsu Orca Flash 4.0 v2). With the 488 nm excitation, emitted fluorescence was split using a 594 nm dichroic (Semrock) before passing through a LP 594 nm filter (Chroma) on camera A and 525/50 nm (Chroma) filter on camera B. This allowed simultaneous detection of Fluo-4 AM signals by camera B at 500-550 nm range and Di-4-ANEPPDHQ signals by camera A for wavelengths longer than 594 nm. With the 589 nm excitation, emitted fluorescence from Mitotracker Red CMXRos was detected on camera A with the same detection range as previous. All data were acquired in an imaging chamber (Okolabs) set to 36°C and 5% humidified CO_2.

For deconvolution, point spread functions (PSFs) were measured using 100 nm Tetraspeck beads on the surface of a 5 mm coverslip. Data were deskewed and deconvolved using LLSpy, a Python interface for processing LLSM data. Deconvolution was performed using a Richardson-Lucy algorithm with 15 iterations with the PSFs generated for each excitation wavelength.

Parasite-associated hots membrane (PAM) plotting

Parasite-erythrocyte interactions were characterized by plotting the amount of surface contact at each timepoint for each event. The analysis was performed using IMARIS (Version 9.7.2, Bitplane) with Tracking module. A surface called 'Erythrocytes' was first created from the erythrocyte membrane channel with smoothing and absolute intensity setting. The threshold was either adjusted automatically or manually, on some occasions, to obtain an almost continuous surface on the erythrocyte of interest while maintaining the original boundary of the cell. Next, a surface called 'All parasites' was created from the parasite channel with smoothing and background subtraction setting. The threshold was adjusted accordingly to achieve reasonable values for parasite surface area (4-9 μm^2) and 0.5 μm seed point value was used to split touching parasites. Next, a masked erythrocyte membrane channel was created from the erythrocyte surface by setting the voxel value inside the surface to 1 and outside the surface to 0. From the 'All parasites' surface, parasites that interact with the erythrocyte were then selected, either by automated tracking or manual selection, and duplicated into individual surfaces called 'Parasite 1', 'Parasite 2', etc. For each parasite, all parts of the surface were selected and then unified and made into a single track. Finally, values of the 'Intensity Sum' from the masked erythrocyte membrane channel and the 'Area' at each timepoint were extracted from each parasite surface and exported to Microsoft Excel. The 'Intensity Sum' values represent the number of voxels in the erythrocyte membrane channel in contact with the parasite surface. The PAM values were then plotted from the Intensity Sum and normalized by the Area.

Airyscan super-resolution microscopy

Synchronised schizonts were purified using Vario MACS CS columns and kept in 1 nM E64 for 4-6 hr. Free merozoites were obtained by passing mature schizonts through a 1.2 μm filter, mixed with red blood cells and incubated for 1 min 30 sec at 37°C and then fixed.

Parasites were fixed with 4% Paraformaldehyde and 0.01% glutaraldehyde for 30 min, permeabilised with 0.1% TX-100 in PBS for 25 min and incubated in blocking solution (2% BSA in PBS) for 1 hr. For testing if PfCSS and PfPTRAMP are surface exposed on merozoites during invasion the permeabilization step was omitted. Following blocking, the samples were incubated with the following primary antibodies diluted in blocking solution: rat anti-HA (Roche 3F10, 1:300), mouse monoclonal 3D8 anti-PfPTRAMP (1:200), mouse monoclonal 8B9 (1:200) [2], rabbit anti-RON4 serum (1:1000), were used. Secondary antibodies labelled with Alexa 488/594 fluorophores were used at 1:1000 dilution. Following the incubation in secondary antibodies, parasites were washed extensively in PBS and mounted on coverslips coated with 1% poly-ethyleneimine with Vectashield containing DAPI (VectorLabs, Australia).

Z-stacks of fluorescently labelled infected red blood cells were imaged with Zeiss LSM880 inverted microscope equipped with a Plan Apochromat 63x/1.4 oil objective with 405, 488, 561 and 594 nm excitations and an Airyscan detector. ImageJ was used for image processing.

Production and analysis of nanobodies

Two Alpacas (both female) were subcutaneously immunized six times 14 days apart with approximately 130 μg of recombinant PfCSS or 100 μg of recombinant PfPTRAMP. The adjuvant used was GERBU FAMA (GERBU Biotechnik GmbH, Heidelberg, Germany) was collected three days after the last immunization for the preparation of lymphocytes. Nanobody library construction was carried out according to established methods [15]. Briefly, alpaca lymphocyte mRNA was extracted and amplified by RT-PCR with specific primers to generate a cDNA library size of 10^8 nanobodies with 80% correct sized nanobody insert. The library was cloned into a pMES4 phagemid vector amplified in *Escherichia coli* TG1 strain and subsequently infected with M13K07 helper phage for recombinant phage expression.

Biopanning for PfPTRAMP and PfCSS nanobodies using phage display was performed as previously described [15]. Phages displaying PfPTRAMP or PfCSS-specific nanobodies were enriched after two rounds of biopanning on 1 mg of immobilized PfPTRAMP or PfCSS protein. After the second round of panning, 95 individual clones were selected for further analyses by ELISA for the presence of PfPTRAMP or PfCSS nanobodies. Positive clones were sequenced

and annotated using the International ImMunoGeneTics database (IMGT) and aligned in Geneious Prime.

Nanobodies were expressed in *E. coli* WK6 cells [16]. Bacteria were grown in Terrific Broth at 37 °C to an OD_{600} of 0.7, induced with 1 mM IPTG and grown overnight at 28 °C for 16 hr. Cell pellets were harvested and resuspended in 20% sucrose, 20 mM imidazole, 150 mM NaCl DPBS and incubated for 15 min on ice. 5 mM EDTA was added and incubated on ice for 20 min. After this incubation, 10 mM $MgCl_2$ was added to prevent EDTA chelation, periplasmic extracts were harvested by centrifugation and the supernatant was loaded onto a 1 ml HisTrap FF column (GE Healthcare). The nanobody was eluted via a linear gradient into 400 mM imidazole, 100 mM NaCl, PBS and buffer exchanged in PBS.

96-well flat-bottomed MaxiSorp plates were coated with 125 nM of recombinant protein as indicated in 50 µL of PBS at room temperature for one hour. All washes were done three times using PBS and 0.1% Tween (DPBS-T) and all incubations were performed for one hour at room temperature. Coated plates were washed and blocked by incubation with 10% skim milk solution. Plates were washed and then incubated with 0 nM - 1000 nM of nanobodies. The plates were washed and incubated with mouse anti-His (Bio-Rad MCA-1396; 1:1000) followed by horseradish peroxidase (HRP)-conjugated goat anti-mouse secondary antibody (MerckMillipore AP124P, 1:1000). After a final wash, 50 µL of azino-bis-3-ethylbenthiazoline-6-sulfonic acid (ABTS liquid substrate; Sigma) was added and incubated in the dark at room temperature and 50 µL of 1% SDS was used to stop the reaction. Absorbance was read at 405 nm and all samples were done in duplicate.

***P. falciparum* schizont supernatant and merozoite preparations and analysis**

Merozoite and supernatant preparations for SDS-PAGE and immunoblot analysis were performed as previously described [7]. Synchronised late trophozoite cultures were passed over LD magnetic columns (Miltenyi Biotech) to remove uninfected erythrocytes. Eluted parasites were adjusted to $5x10^6$ schizonts/ml and 150 µl added per well of a 96-well-flat bottomed culture dish. The assay dishes were further cultured for 16 hr and a representative well smeared for Giemsa staining, to ensure either that rupture had occurred normally (control well) or that rupture had been blocked when inhibitors were added. Parasites from each condition were spun at 10,000g/10 min to collect the merozoite pellet and supernatant fractions. Proteins from both

fractions were extracted with Reducing sample buffer and separated on 4-12% or 3-8% acrylamide gels (NuPAGE, Invitrogen). When inhibitors WM4 at 40 nM and WM382 at 2.5 nM final concentrations a control dish without any protease inhibitor was also included. Parasites were eluted from columns with complete RPMI 1640 culture medium to which the appropriate inhibitor at the same concentration had been added.

Expression and purification of PfCSS, PfPTRAMP, PTRAMP-CSS heterodimer, PfRipr, CyRPA and PfRh5

The gene for the PfPMX cleaved ectodomain of PfPTRAMP (residues 42 to 309) was subcloned into a modified pTRIEX2 vector with N-terminal SUMO and Flag tags followed by a TEV protease cleavage site. One potential N-linked glycosylation site at Asn195 was removed by mutation of Thr197 to Ala. The construct was expressed in Sf21 insect cells and secreted into the medium as a soluble protein. The supernatant was purified by ANTI-FLAG M2 Affinity Gel (Merck) and size exclusion chromatography (S200 Increase 10/300 GL, Cytiva). Fractions containing PfPTRAMP were pooled and cleaved with TEV protease for 16 h at 4 °C. His-tagged TEV was removed via NiNTA Agarose resin (Qiagen) and PfPTRAMP was further purified via another size exclusion chromatography (S200 Increase 10/300 GL, Cytiva). For biopanning anti-PfPTRAMP nanobodies and their kinetic characterization, a PfPTRAMP (42-309) construct with a C-terminal Avitag was generated and specifically biotinylated [17]. In addition, a PfPTRAMP construct comprised of residues 25 to 309 with a C-terminal His-tag was used for BLI binding studies to PfCSS, however the purification was the same.

The gene for PfCSS (residues 20 to 290) was subcloned into a modified pTRIEX2 vector with a C-terminal Flag tag preceded by a TEV protease cleavage site. The construct was expressed in Sf21 insect cells and purified similarly to PfPTRAMP. The construct used for the alpaca immunization had no potential N-glycosylation sites mutated and was therefore glycosylated. The construct used in binding and crystallization studies had one glycan removed at Asn261, by mutation of Thr263 to Ala.

To generate disulfide-linked PTRAMP-CSS, PfPTRAMP (42-309) and PfCSS (20-290) constructs were co-expressed in Sf21 insect cells and purified in a similar manner to PfPTRAMP described above. The PTRAMP-CSS construct used to test D2 nanobody glycan dependency had four out of five potential N-linked glycan sites at Asn74, Asn192, Asn234 and

Asn261 removed via mutation of the glycan site Thr or Ser to Ala. Mutation of the glycan at Asn283 led to no expression and so was not included. To test binding of nanobodies to PTRAMP-CSS, a biotinylated PTRAMP-CSS protein was generated using the PfPTRAMP (42-309) construct with a C-terminal Avitag.

The gene for PfRipr (residues 20 to 1086) was subcloned into pACGP67a with a C-terminal His-tag. The construct was expressed in Sf21 cells and secreted into the medium as soluble protein. The supernatant was dialysed into 20 mM Tris pH 8, 150mM NaCl. Imidazole was added to 10mM final concentration and PfRipr was purified by NiNTA Agarose (Qiagen) and eluted in 20mM Tris pH 8, 150 mM NaCl, 500 mM Imidazole. The sample was further purified via size exclusion chromatography, using a S200 Increase 10/300 GL (Cytiva).

The gene for CyRPA (residues 29 to 362) was subcloned into a modified pcDNA3.4-TOPO plasmid with an N-terminal IL-2 signal sequence and a C-terminal Flag preceded by a TEV protease cleavage site. Three potential N-linked glycosylation sites at Asn145, Asn322 and Asn338 were removed by mutation of the glycan site Thr or Ser residues to Ala. The construct was expressed via transient transfection of Expi293F cells and soluble protein was purified from the culture medium in a similar manner to PfPTRAMP described above.

The gene for PMX cleaved PfRh5 (residues 145 to 526) was subcloned into pACGP67a with a C-terminal C-tag. Three potential N-linked glycosylation sites as Asn214, Asn284 and Asn297 were removed by mutation of Thr or Ser residues to Ala. The construct was expressed in Sf21 cells and secreted into the medium as soluble protein. The supernatant was purified by CaptureSelect C-tagXL Affinity Matrix (Thermofisher) and eluted with 20 mM Tris pH 7.5, 2 M $MgCl_2$. The sample was further purified via size exclusion chromatography, using a S200 Increase 10/300 GL (Cytiva).

Biolayer Interferometry studies

Biolayer interferometry experiments were conducted at 25 °C to determine the affinity and epitope bins of selected proteins and nanobodies for PfCSS. For protein-protein binding kinetic studies, either PfRipr or PfPTRAMP were diluted into kinetics buffer (PBS, pH 7.4, 0.1% (w/v) BSA, 0.02% (v/v) Tween-20) at 20 μg/mL and immobilized onto Anti-Penta-His (His1K) biosensors (Sartorius). Following a 60 s baseline step, biosensors were dipped into wells

containing twofold dilution series of either PTRAMP-CSS or PfCSS. Sensors were then dipped back into kinetics buffer to monitor the dissociation rate. For nanobody-PfCSS binding kinetic studies, nanobodies were diluted in kinetics buffer to 5 µg/mL and immobilized onto Ni-NTA (NTA) biosensors (Sartorius). Following a 60 s baseline step, biosensors were dipped into wells containing twofold dilution series of either PTRAMP-CSS or PfCSS. Sensors were then dipped back into kinetics buffer to monitor the dissociation rate. For nanobody-PfPTRAMP binding kinetic studies, biotinylated PfPTRAMP or PTRAMP-CSS were immobilized onto High Precision Streptavidin (SAX) biosensors (Sartorius). Following a 60 s baseline step, biosensors were dipped into wells containing twofold dilution series of anti-PfPTRAMP nanobodies.

For competition studies of the anti-PfCSS nanobodies, nanobodies were first diluted in kinetics buffer to 5 µg/mL and immobilized onto Ni-NTA (NTA) biosensors (Sartorius). Following a 30 s baseline step, biosensors were dipped into wells containing a negative control nanobody that does not bind the proteins under analysis to quench the sensors. Following another 30 s baseline step, biosensors were dipped into either PfCSS or PTRAMP-CSS. Following a final 30 s baseline step, biosensors were then dipped into a secondary nanobody or PfRipr to assess competition. Due to the moderate affinity of the anti-PfPTRAMP nanobodies, a premix format was employed. Nanobodies or PfRipr were first diluted to 10 µg/mL and immobilized onto Anti-Penta-His (His1K) biosensors. Following a 30 s baseline step, biosensors were dipped into wells containing a negative control nanobody that does not bind the proteins under analysis to quench the sensors. Following another 30 s baseline step, biosensors were then dipped into PTRAMP-CSS pre-incubated with a 10-fold molar excess of competing secondary nanobody to assess competition.

Kinetics and competition data were analyzed using Sartorius' Data Analysis software 11.0. Kinetic curves were fitted to a 1:1 binding model. Mean kinetic constants reported are the result of two independent experiments. Data presented in Fig. S4 represent the percent of competing nanobody or PfRipr binding compared to the maximum competing nanobody response.

Growth inhibition assays with nanobodies and *P. falciparum* parasites

One-cycle growth inhibition assays were performed largely as described previously [18]. Trophozoite stage parasites at 0.4% parasitemia were grown in a 50 µl culture volume at 2% hematocrit in 96 well round bottom microtitre plates (Falcon) with two-fold dilutions of each nanobody. After incubation for 48 hr, each well was fixed at room temperature for 30 min with

50 µl of 0.25% glutaraldehyde (ProSciTech) diluted in human tonicity PBS. Following centrifugation at 1,200 rpm for 2 min, supernatants were discarded, and parasites stained with 50 µl SYBR Green (Invitrogen) diluted in PBS. The parasitemia of each well was determined by counting 100,000 cells by flow cytometry using an Attune NxT Flow Cytometer (ThermoFisher). Growth was expressed as a percentage of the parasitemia obtained using a non-immune IgG or vehicle control. All samples were tested in triplicate and standard error of the mean calculated.

Flow-cytometric analysis of erythrocyte binding and Ca^{2+} flux

Erythrocyte binding assays were performed as described with some minor changes [19]. Briefly, O+ erythrocytes were made up to a final density of ~1 x 10^7 cells/mL in 1X PBS + 1% (w/v) BSA (PBS/BSA). All incubations were in 100 µL volume and washes in PBS/BSA at room temperature unless otherwise stated. Recombinant proteins were used at 400 nM final concentration. Complexes were made at equimolar ratios of 400 nM and incubated at room temperature for 1 hr for complex formation. Each sample was prepared using 100 µL of resuspended erythrocytes which were centrifuged at 2,000x *g* for 1 min, supernatant removed, and the pre-incubated protein complexes or PBS/BSA added. After a 1 hr incubation on a roller, cells were centrifuged at 2,000x *g* and washed once before a primary antibody was added. All antibodies were used at a final concentration of 0.2 mg/mL. After incubation for 1 hr, cells were washed once, and an Alexa-Flour 488 (Life Technologies) conjugated secondary antibodies (anti-mouse, anti-rabbit, or anti-rat) added at 1:100 dilution. Cells were washed twice in PBS and resuspended in 600 µL followed by analysis with a LSRII flow cytometer (BD Life Sciences). Fifty-thousand events were recorded, and results analysed using FlowJo™ v10.7 Software (BD Life Sciences). For quantitation, the background signal of erythrocytes incubated with only primary and secondary antibodies was subtracted from the signal of erythrocytes incubated with recombinant protein and relevant primary and secondary antibodies, divided by the total number of events and then multiplied by 100 to achieve a percentage binding value. Statistical analysis was performed in Prism 9 (GraphPad) using an ordinary one-way ANOVA with multiple comparisons.

Analysis of Ca^{2+} flux across the erythrocyte membrane was performed as described previously [19]. PCRCR was prepared at 8 µM and diluted into the erythrocyte suspension to 4 µM to test stimulation of Ca^{2+} flux. An LSRII flow cytometer (BD Life Sciences) was used for analysing

samples and the results analysed in FlowJo™ v10.7 Software (BD Life Sciences) using the kinetics package.

3-dimensional structure determination of PfCSS-nanobody complexes

For crystallization studies, PTRAMP-CSS and PfCSS alone were mixed with D2 and H2, respectively, in a 1:2 molar ratio and excess nanobody was purified away via size exclusion chromatography (Superdex 200 Increase 10/300 GL, Cytiva). Complexes were then concentrated to 5 mg/mL and mixed 1:1 with mother liquor and setup in hanging or sitting drop crystallization experiments. D2-PTRAMP-CSS crystallized in 1.6 M ammonium sulfate, 0.1 M sodium chloride, 0.1 M sodium HEPES pH 7.5, after one month, and were cryoprotected in 15% (v/v) ethylene glycol. H2-PfCSS crystallized in 0.1 M bis-tris-propane pH 6.0, 17.5% (v/v) PEG3350, 0.2 M sodium malonate, in 24 h, and were cryoprotected in 15% (v/v) ethylene glycol. Data were collected at the MX2 beamline at the Australian Synchrotron, processed and merged using XDS [20] and Aimless [21]. The positions of the H2 nanobodies in the H2-PfCSS crystal structure were first determined by molecular replacement using the structure of nanobody VHH-α204 from 5HVG with its CDR3 removed [22]. This solution was then used to build the two PfCSS structures present in the asymmetric unit via AutoBuild [23]. This PfCSS structure was then used as a model for molecular replacement in the low-resolution crystal structure of D2-CSS, along with VHH-72 from 6WAQ [24]. PfPTRAMP was not present in the D2-PTRAMP-CSS crystal structure. Presumably, PfPTRAMP and PfCSS dissociated during crystallization, and only D2-CSS crystallized after 1 month in the high salt crystallization condition. Refinement of the structures was carried out using phenix.refine [25] and iterations of refinement using Coot [26].

Data availability

The crystal structures reported in this manuscript have been deposited in the Protein Data Bank, www.rcsb.org (PDB ID codes 7UNY, 7UNZ). The mass spectrometry proteomics data have been deposited to the ProteomeXchange Consortium via the PRIDE [27] partner repository with the dataset identifier PXD (reviewer token: Username: reviewer@ebi.ac.uk Password:).

Statistical Analysis

Data processing and analysis were performed using R (version 4.1.2). The false hits, including contaminants, reverse proteins, and proteins identified by site were removed. Only proteins that

were quantified in at least 67% of replicates in at least one condition were kept. The protein intensities were log_2-transformed. Missing values were imputed by using Missing Not At Random (MNAR) method. This was achieved by substituting 'NAs' with numbers that were drawn from a normal distribution with a mean that is left-shifted from the sample mean by 1.8 standard deviation with a width of 0.3 [28].

Statistical analysis for protein binding to erythrocytes using FACS was performed using GraphPad Prism v 9.3.1 for Mac. Data are presented as mean and standard error of the mean (SEM). For GIA curves EC_{50} was determined through a four-parameter logistic regression with the Top parameter constrained to 100% GIA.

Statistical analysis was performed in Prism 9 (GraphPad) using an ordinary one-way ANOVA with multiple comparisons.

The protein differential expression and enrichment analysis data were normalized using RUVIIIC [29]. The optimum k value used to remove the unwanted variation was determined based on PCA, RLE and p-value distribution plots. The R-package limma [30] (v. 3.50.1) was used to perform the differential analysis. A protein was determined to be significantly differentially expressed if the false discovery rate (FDR) adjusted p-value was ≤ 0.05. R-packages; ggplot2 (v. 3.3.5) was used to visualise the results.

References

1 Trager, W. & Jensen, J. B. Cultivation of malarial parasites. *Nature* **273**, 621-622 (1978).

2 Chen, L. *et al.* Structural basis for inhibition of erythrocyte invasion by antibodies to *Plasmodium falciparum* protein CyRPA. *eLife* **6**, doi:10.7554/eLife.21347 (2017).

3 Chen, L. *et al.* An EGF-like protein forms a complex with PfRh5 and is required for invasion of human erythrocytes by *Plasmodium falciparum*. *PLoS Pathog.* **7**, e1002199, doi:10.1371/journal.ppat.1002199 (2011).

4 Healer, J. *et al.* Neutralising antibodies block the function of Rh5/Ripr/CyRPA complex during invasion of *Plasmodium falciparum* into human erythrocytes. *Cell Microbiol.* **21**, e13030, doi:10.1111/cmi.13030 (2019).

5 Richard, D. *et al.* Interaction between *Plasmodium falciparum* apical membrane antigen 1 and the rhoptry neck protein complex defines a key step in the erythrocyte invasion process of malaria parasites. *J. Biol. Chem.* **285**, 14815-14822, doi:10.1074/jbc.M109.080770 (2010).

6 Knuepfer, E., Napiorkowska, M., van Ooij, C. & Holder, A. A. Generating conditional gene knockouts in *Plasmodium* - a toolkit to produce stable DiCre recombinase-

expressing parasite lines using CRISPR/Cas9. *Scientific reports* **7**, 3881, doi:10.1038/s41598-017-03984-3 (2017).

7 Favuzza, P. *et al.* Dual Plasmepsin-Targeting Antimalarial Agents Disrupt Multiple Stages of the Malaria Parasite Life Cycle. *Cell Host Microbe* **27**, 642-658 e612, doi:10.1016/j.chom.2020.02.005 (2020).

8 Wisniewski, J. R., Zougman, A., Nagaraj, N. & Mann, M. Universal sample preparation method for proteome analysis. *Nat Methods* **6**, 359-362, doi:10.1038/nmeth.1322 (2009).

9 Delconte, R. B. *et al.* CIS is a potent checkpoint in NK cell-mediated tumor immunity. *Nat Immunol* **17**, 816-824, doi:10.1038/ni.3470 (2016).

10 Cox, J. *et al.* Andromeda: a peptide search engine integrated into the MaxQuant environment. *J Proteome Res* **10**, 1794-1805, doi:10.1021/pr101065j (2011).

11 Lambros, C. & Vanderberg, J. P. Synchronization of *Plasmodium falciparum* erythrocytic stages in culture. *Journal of Parasitology* **65**, 418-420 (1979).

12 Volz, J. C. *et al.* Essential Role of the PfRh5/PfRipr/CyRPA Complex during *Plasmodium falciparum* Invasion of Erythrocytes. *Cell Host Microbe* **20**, 60-71, doi:10.1016/j.chom.2016.06.004 (2016).

13 Geoghegan, N. D. *et al.* 4D analysis of malaria parasite invasion offers insights into erythrocyte membrane remodeling and parasitophorous vacuole formation. *Nat. Commun.* **12**, 3620, doi:10.1038/s41467-021-23626-7 (2021).

14 Chen, B. C. *et al.* Lattice light-sheet microscopy: imaging molecules to embryos at high spatiotemporal resolution. *Science* **346**, 1257998, doi:10.1126/science.1257998 (2014).

15 Nunes-Silva, S. *et al.* Llama immunization with full-length VAR2CSA generates cross-reactive and inhibitory single-domain antibodies against the DBL1X domain. *Scientific reports* **4**, 7373, doi:10.1038/srep07373 (2014).

16 Pardon, E. *et al.* A general protocol for the generation of Nanobodies for structural biology. *Nat. Protoc.* **9**, 674-693, doi:10.1038/nprot.2014.039 (2014).

17 Fairhead, M. & Howarth, M. Site-specific biotinylation of purified proteins using BirA. *Methods Mol. Biol.* **1266**, 171-184, doi:10.1007/978-1-4939-2272-7_12 (2015).

18 Healer, J. *et al.* Vaccination with conserved regions of erythrocyte-binding antigens induces neutralizing antibodies against multiple strains of *Plasmodium falciparum. PLoS One* **8**, e72504, doi:10.1371/journal.pone.0072504 (2013).

19 Wong, W. *et al.* Structure of *Plasmodium falciparum* Rh5-CyRPA-Ripr invasion complex. *Nature* **565**, 118-121, doi:10.1038/s41586-018-0779-6 (2019).

20 Kabsch, W. XDS. *Acta crystallographica. Section D, Biological crystallography* **66**, 125-132, doi:10.1107/S0907444909047337 (2010).

21 Evans, P. R. & Murshudov, G. N. How good are my data and what is the resolution? *Acta Crystallogr. D. Biol. Crystallogr.* **69**, 1204-1214, doi:10.1107/S0907444913000061 (2013).

22 Zhou, X. *et al.* Elucidation of the molecular mechanisms of two nanobodies that inhibit thrombin-activatable fibrinolysis inhibitor activation and activated thrombin-activatable fibrinolysis inhibitor activity. *J. Thromb. Haemost.* **14**, 1629-1638, doi:10.1111/jth.13381 (2016).

23 Terwilliger, T. C. *et al.* Iterative model building, structure refinement and density modification with the PHENIX AutoBuild wizard. *Acta Crystallogr. D Biol. Crystallogr.* **64**, 61-69, doi:10.1107/S090744490705024X (2008).

24 Wrapp, D. *et al.* Structural Basis for Potent Neutralization of Betacoronaviruses by Single-Domain Camelid Antibodies. *Cell* **181**, 1004-1015 e1015, doi:10.1016/j.cell.2020.04.031 (2020).

25 Adams, P. D. *et al.* PHENIX: a comprehensive Python-based system for macromolecular structure solution. *Acta Cryst. D. Biol. Crystallogr.* **66**, 213-221 (2010).
26 Emsley, P. & Cowtan, K. Coot: model-building tools for molecular graphics. *Acta Cryst. D. Biol. Crystallogr.* **60**, 2126-2132 (2004).
27 Perez-Riverol, Y. *et al.* The PRIDE database and related tools and resources in 2019: improving support for quantification data. *Nucleic Acids Res.* **47**, D442-D450, doi:10.1093/nar/gky1106 (2019).
28 Lazar, C., Gatto, L., Ferro, M., Bruley, C. & Burger, T. Accounting for the Multiple Natures of Missing Values in Label-Free Quantitative Proteomics Data Sets to Compare Imputation Strategies. *Journal of proteome research* **15**, 1116-1125, doi:10.1021/acs.jproteome.5b00981 (2016).
29 Poulos, R. C. *et al.* Strategies to enable large-scale proteomics for reproducible research. *Nat Commun* **11**, 3793, doi:10.1038/s41467-020-17641-3 (2020).
30 Ritchie, M. E. *et al.* limma powers differential expression analyses for RNA-sequencing and microarray studies. *Nucleic Acids Res.* **43**, e47, doi:10.1093/nar/gkv007 (2015).
