# Supplementary material for: PCRCR complex is essential for invasion of human erythrocytes by Plasmodium falciparum
Source: Nat Microbiol. 2022 Nov 17;7(12):2039–53. doi: 10.1038/s41564-022-01261-2 (PMC9712106; doi:10.1038/s41564-022-01261-2)
Supplement: Supplementary file 2 — Reporting Summary [file 41564_2022_1261_MOESM2_ESM.pdf]

nature portfolio

Corresponding author(s): Alan F. Cowman

Last updated by author(s): Sep 9, 2022

Reporting Summary

Nature Portfolio wishes to improve the reproducibility of the work that we publish. This form provides structure for consistency and transparency in reporting. For further information on Nature Portfolio policies, see our Editorial Policies and the Editorial Policy Checklist.

Statistics

For all statistical analyses, confirm that the following items are present in the figure legend, table legend, main text, or Methods section.

n/a	Confirmed	
☐	☒	The exact sample size (*n*) for each experimental group/condition, given as a discrete number and unit of measurement
☐	☒	A statement on whether measurements were taken from distinct samples or whether the same sample was measured repeatedly
☐	☒	The statistical test(s) used AND whether they are one- or two-sided<br>*Only common tests should be described solely by name; describe more complex techniques in the Methods section.*
☒	☐	A description of all covariates tested
☒	☐	A description of any assumptions or corrections, such as tests of normality and adjustment for multiple comparisons
☐	☒	A full description of the statistical parameters including central tendency (e.g. means) or other basic estimates (e.g. regression coefficient) AND variation (e.g. standard deviation) or associated estimates of uncertainty (e.g. confidence intervals)
☐	☒	For null hypothesis testing, the test statistic (e.g. *F*, *t*, *r*) with confidence intervals, effect sizes, degrees of freedom and *P* value noted<br>*Give P values as exact values whenever suitable.*
☒	☐	For Bayesian analysis, information on the choice of priors and Markov chain Monte Carlo settings
☒	☐	For hierarchical and complex designs, identification of the appropriate level for tests and full reporting of outcomes
☒	☐	Estimates of effect sizes (e.g. Cohen's *d*, Pearson's *r*), indicating how they were calculated

Our web collection on statistics for biologists contains articles on many of the points above.

Software and code

Policy information about availability of computer code

Data collection	For Lattice Light Sheet imaging data was deskewed and deconvolved using LLSpy, a Python interface.<br>Deconvolution was performed using a Richardson-Lucy algorithm using the PSFs generated for each excitation wavelength. Compass Hystar 5.1.
Data analysis	ImageJ version 2.3.0/1.53n. MaxQuant (version 1.5.8.3 or 1.6.17). IMARIS (Version 9.7.2, Bitplane). Octet Data Analysis software 11.0 (Sartorius). FlowJoTM v10.7. R (version 4.1.2). GraphPad Prism v 9.3.1. R-package limma (v. 3.50.1). ggplot2 (v. 3.3.5). Crystallographic data were processed and merged using XDS(VERSION Nov 1, 2016, VERSION Jan 26, 2018) and Aimless (0.7.4). Structures were determined by molecular replacement using Phaser (2.8.3). The H2-PfCSS crystal structure was partially built using AutoBuild. Refinement of the structures was carried out using Phenix (1.19.2) and iterations of refinement using Coot (0.8.9.2).

For manuscripts utilizing custom algorithms or software that are central to the research but not yet described in published literature, software must be made available to editors and reviewers. We strongly encourage code deposition in a community repository (e.g. GitHub). See the Nature Portfolio guidelines for submitting code & software for further information.

Data

Policy information about availability of data

All manuscripts must include a data availability statement. This statement should provide the following information, where applicable:

- Accession codes, unique identifiers, or web links for publicly available datasets
- A description of any restrictions on data availability
- For clinical datasets or third party data, please ensure that the statement adheres to our policy

The crystal structures reported in this manuscript have been deposited in the Protein Data Bank, www.rcsb.org (PDB ID codes 7UNY, 7UNZ). The mass spectrometry

proteomics data have been deposited to the ProteomeXchange Consortium via the PRIDE 59 partner repository with the dataset identifier PXD (reviewer token: Username: reviewer@ebi.ac.uk Password:). P. falciparum sequences were derived from PlasmoDB (https://plasmodb.org/plasmo/app). The datasets generated during and/or analysed during the current study are available from the corresponding author on reasonable request.

Field-specific reporting

Please select the one below that is the best fit for your research. If you are not sure, read the appropriate sections before making your selection.

- [x] Life sciences
- [ ] Behavioural & social sciences
- [ ] Ecological, evolutionary & environmental sciences

For a reference copy of the document with all sections, see nature.com/documents/nr-reporting-summary-flat.pdf

Life sciences study design

All studies must disclose on these points even when the disclosure is negative.

Sample size	No statistical method was used to predetermine sample size. Instead, sample sizes were chosen according to best practices in the field and previous studies (Wong et al. Nature 2019, Geoghegan et al. Nat Comms 2021, Ragotte et al. Nat Comms 2022).
Data exclusions	No data were excluded.
Replication	Monitoring of P. falciparum parasitemia of the inducible knockdowns was performed in duplicate in two independent experiments. The number of invasion events recorded by lattice light sheet microscopy is detailed in Supplementary Table 1. BLI kinetic experiments were performed at least twice. Erythrocyte binding assays were performed in triplicate on separate days. All growth inhibition assays were performed in three independent experiments, with data points representing the mean from one experiment, performed in triplicate, except in Extended Data Fig 7a, which was performed once in triplicate. All attempts at replication were successful.
Randomization	Randomization was not relevant to this study as no subjective judgements were required about which data to include, exclude or measure.
Blinding	The investigators were not blinded to the group allocation during the experiment and/or when assessing the outcome.

Reporting for specific materials, systems and methods

We require information from authors about some types of materials, experimental systems and methods used in many studies. Here, indicate whether each material, system or method listed is relevant to your study. If you are not sure if a list item applies to your research, read the appropriate section before selecting a response.

Materials & experimental systems

n/a	Involved in the study	
[ ]	[x]	Antibodies
[ ]	[x]	Eukaryotic cell lines
[x]	[ ]	Palaeontology and archaeology
[ ]	[x]	Animals and other organisms
[x]	[ ]	Human research participants
[x]	[ ]	Clinical data
[x]	[ ]	Dual use research of concern

Methods

n/a	Involved in the study	
[x]	[ ]	ChIP-seq
[ ]	[x]	Flow cytometry
[x]	[ ]	MRI-based neuroimaging

Antibodies

Antibodies used	Antibodies and monoclonal antibodies were raised in rabbits and mice and all procedures approved by the Walter and Eliza Hall Institute of Medical Research Animal Ethics Committee. Immunization and handling of the alpaca for scientific purposes was approved by Agriculture Victoria, Wildlife and Small Institutions Animal Ethics Committee, project approval No. 26-17.<br>In this study, we used the following antibodies: rat mAb, anti-HA (Roche 3F10, Cat.: 11867423001, Lot: 47877600); mouse mAbs, 1D9 and 3D8 anti-PfPTRAMP (this study), rat mAb 2D2 anti-PfCSS (this study), mouse mAbs 5B12, 7A6 and 8B9 anti-CyRPA (Chen et al, Elife 2017), 5A9 and 6H2 PfRh5 (Chen et al, Plos Path 2011), mouse mAb 1G12 anti-Ripr (Healer et al, Cell Microbiol 2019), rabbit anti-RON4 polyclonal (Richard et al, JBC, 2010); rat pAb KM81 anti-PfCSS (this study); rabbit pAb R1541 anti-Ripr (Healer et al, Cell Microbiol 2019).<br><br>The mAbs 1D9 and 3D8 that bound to PfPTRAMP were raised in mice at the WEHI Antibody Facility, by immunising with recombinant PfPTRAMP expressed and purified from insect cells. Briefly, PfPTRAMP (N25-K309) between the end of the signal sequence and the start of the transmembrane domain was recodoned for insect cell expression (Genscript) and cloned into an insect cell expression vector bearing an N-terminal gp67 signal peptide, a SUMO tag, a FLAG tag and a tobacco etch virus (TEV) protease cleavage site.<br><br>The 2D2 mAb and pAb KM81 that bound to PfCSS were made in rats, at the WEHI Antibody Facility, by immunising with PfCSS recombinant protein expressed and purified from insect cells. PfCSS (Q21-K290) after the end of the signal sequence was recodoned for insect cell expression (Genscript) and cloned into an expression vector bearing an N-terminal gp67 signal peptide and a C-terminal

fusion tag comprising a TEV site and a FLAG tag.

The following secondary antibodies labelled with Alexa 488/594 fluorophores (Life Technologies) and HRP antibodies were used: chicken anti-mouse 594 (Cat.: A21201, Lot: 42099A), donkey anti-rat 488 (Cat.: A21208, Lot: 2310102), chicken anti-rabbit 594 (Cat.: A21442, Lot: 2110863), goat anti-mouse 488 (Cat.: A11001), goat anti-rabbit (Cat.: A11008), Peroxidase Affinity Pure Goat Anti-Human IgG (H+L) (Cat No. 109-035-088, Jackson Immuno Research).

Validation

Rat mAb, anti-HA (Roche 3F10, Cat.: 11867423001) validated by the supplier by western blot.
Chicken anti-mouse 594 (Cat.: A21201, Lot: 42099A) validated by the supplier by IFA.
Donkey anti-rat 488 (Cat.: A21208, Lot: 2310102) validated by the supplier by IFA.
Chicken anti-rabbit 594 (Cat.: A21442, Lot: 2110863) validated by the supplier by IFA.
Goat anti-mouse 488 (Cat.: A11001) validated by the supplier by IFA and Flow cytometry.
Goat anti-rabbit 488 (Cat.: A11008) validated by the supplier by IFA and Flow cytometry.
Peroxidase Affinity Pure Goat Anti-Human IgG (H+L) (Cat No. 109-035-088) validated by the supplier by western blot and ELISA.

anti-PfPTRAMP mAb 1D9 was validated by western blot in Figure 4a and Extended Data Figures 2 and 4. anti-PfPTRAMP mAb 3D8 was validated by IFA in Figure 3 and flow cytometry in Figure 4g.

anti-PfCSS mAb 2D2 was validated by western blot in Extended Data Figures 2 and 4. Rat pAb KM81 anti-PfCSS was validated by flow cytometry in Figure 4g.

Anti-CyRPA mAb 5B12 was validated in Chen et al, Elife 2017 by GIA and in this paper by flow cytometry. Anti-CyRPA mAb 8B9 from Chen et al, Elife 2017 was validated in this paper by IFA. Anti-CyRPA mAb 7A6 was validated in Chen et al, Elife 2017 and this paper by western blot.

Anti-Rh5 mAb 5A9 was validated in Chen et al, Plos Path 2011 by western blot and in this paper by flow cytometry in Figure 4g. Anti-Rh5 mAb 6H2 was validated in Chen et al, Plos Path 2011 and this paper by western blot (Extended Data Figure 2).

Anti-Ripr mAb 1G12 was validated in Healer et al, Cell Microbiol 2019 by western blot, SPR and GIA and in this paper by western blot in Extended Data Figure 2 and GIA in Figure 5b. Anti-Ripr polyclonal Ab R1541 was validated in Healer et al, Cell Microbiol 2019 by western blot and in this paper by flow cytometry in Figure 4g.

Anti-RON4 polyclonal Ab was validated in Richard et al, JBC 2010 by western blot and IFA and in this paper by IFA in Figure 3.

Eukaryotic cell lines

Policy information about cell lines

Cell line source(s)

3D7 and CS2 P. falciparum lines
Sf21 cells (ThermoFisher Scientific)
Expi293F™ cells (Thermofisher Scientific)
O+ erythrocyte (Australian red-cross bloodbank, South Melbourne, Australia)

Authentication

The P. falciparum lines are periodically sequenced for other projects. This serves as an authentication that they are the expected versions of the P. falciparum lines. Sf21 and Expi293F™ cell lines were purchased or obtained with the certificate of analysis.

Mycoplasma contamination

All cells lines are tested periodically for Mycoplasma infection and were negative.

Commonly misidentified lines
(See ICLAC register)

N/A

Animals and other organisms

Policy information about studies involving animals; ARRIVE guidelines recommended for reporting animal research

Laboratory animals

Two female Balb/C mice received their first immunisation at 8-9 weeks of age. Two female Wistar rats received their first immunisation at 8 weeks of age. Animals were housed in open top cages with irritated feed, autoclaved bedding, were checked daily, dark/light cycle 12 hrs - 7pm-7am dark - 7am-7pm light, temperature set to 21C, ranging from 18-24C, humidity approximately 40% but uncontrolled.

Two female alpacas were immunized with recombinant PfPTRAMP and PfCSS for the generation of nanobodies.

Wild animals

No wild animals were used in this study.

Field-collected samples

No field-collected samples were used in this study.

Ethics oversight

Antibodies and monoclonal antibodies were raised in rats and mice and all procedures approved by the Walter and Eliza Hall Institute of Medical Research Animal Ethics Committee. Immunization and handling of the alpaca for scientific purposes was approved by Agriculture Victoria, Wildlife and Small Institutions Animal Ethics Committee, project approval No. 26-17.

Note that full information on the approval of the study protocol must also be provided in the manuscript.

Flow Cytometry

Plots

Confirm that:

☒ The axis labels state the marker and fluorochrome used (e.g. CD4-FITC).

☒ The axis scales are clearly visible. Include numbers along axes only for bottom left plot of group (a 'group' is an analysis of identical markers).

☒ All plots are contour plots with outliers or pseudocolor plots.

☒ A numerical value for number of cells or percentage (with statistics) is provided.

Methodology

Sample preparation	Erythrocyte binding assays were performed as described with some minor changes 30. Briefly, O+ erythrocytes were made up to a final density of ~1 x 107 cells/mL in 1X PBS + 1% (w/v) BSA (PBS/BSA). All incubations were in 100 µL volume and washes in PBS/BSA at room temperature unless otherwise stated. Recombinant proteins were used at 400 nM final concentration. Complexes were made at equimolar ratios of 400 nM and incubated at room temperature for 1 hr for complex formation. Each sample was prepared using 100 µL of resuspended erythrocytes which were centrifuged at 2,000x g for 1 min, supernatant removed, and the pre-incubated protein complexes or PBS/BSA added. After a 1 hr incubation on a roller, cells were centrifuged at 2,000x g and washed once before a primary antibody was added. All antibodies were used at a final concentration of 0.2 mg/mL. After incubation for 1 hr, cells were washed once, and an Alexa-Flour 488 (Life Technologies) conjugated secondary (anti-mouse, anti-rabbit, or anti-rat) added at 1:100 dilution. Cells were washed twice in PBS and resuspended in 600 µL followed by analysis with a LSRII flow cytometer (BD Life Sciences). Fifty-thousand events were recorded, and results analysed using FlowJoTM v10.7 Software (BD Life Sciences). For quantitation, the background signal of erythrocytes incubated with only primary and secondary antibodies was subtracted from the signal of erythrocytes incubated with recombinant protein and relevant primary and secondary antibodies, divided by the total number of events and then multiplied by 100 to achieve a percentage binding value. Statistical analysis was performed in Prism 9 (GraphPad) using an ordinary one-way ANOVA with multiple comparisons.<br><br>Analysis of Ca2+ flux across the erythrocyte membrane was performed as described previously 30. PCRCR was prepared at 8 µM and diluted into the erythrocytes to 4 µM to test stimulation of Ca2+ flux. An LSRII flow cytometer (BD Life Sciences) was used for analysing samples and the results analysed in FlowJoTM v10.7 Software (BD Life Sciences) using the kinetics package.
Instrument	LSRII flow cytometer (BD Life Sciences).
Software	FlowJoTM v10.7 Software (BD Life Sciences) using the kinetics package.
Cell population abundance	N/A
Gating strategy	The erythrocyte population was gated with SSC-A and FSC-A, then doublets were excluded using FSC-H and FSC-A. For determining complex binding to erythrocytes, a cutoff of >1,000 was used. Gating was performed in an identical manner for all other antibody and antigen combinations.

☒ Tick this box to confirm that a figure exemplifying the gating strategy is provided in the Supplementary Information.
