# Supplementary figures and images for: PCRCR complex is essential for invasion of human erythrocytes by Plasmodium falciparum
Source: Nat Microbiol. 2022 Nov 17;7(12):2039–53. doi: 10.1038/s41564-022-01261-2 (PMC9712106; doi:10.1038/s41564-022-01261-2)

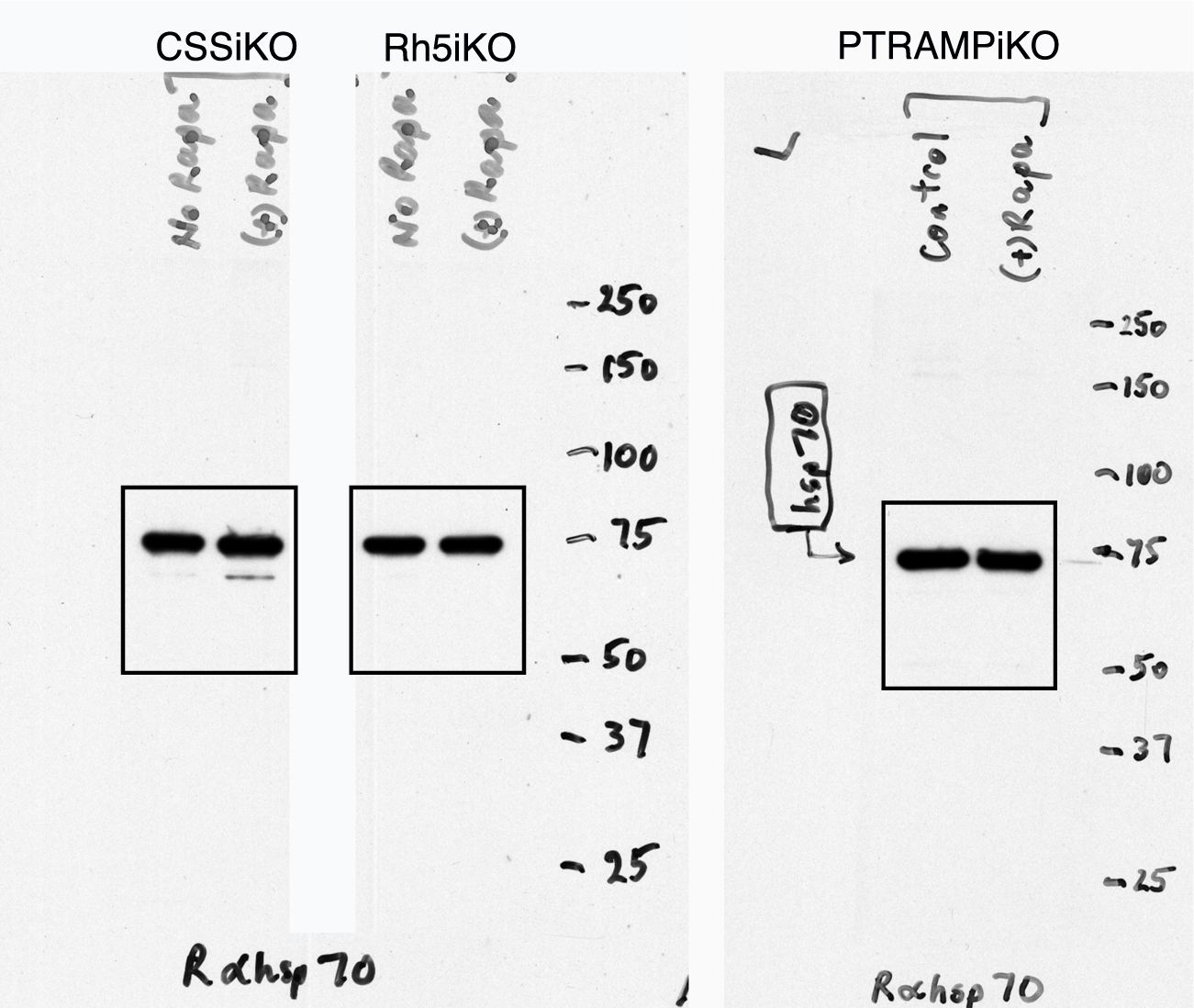

Supplement: Source Data Fig. 1a–c — Unprocessed western blots. [file 41564_2022_1261_MOESM5_ESM.tif]
